# Using Synthetic Mouse Spike-In Transcripts to Evaluate RNA-Seq Analysis Tools

**DOI:** 10.1371/journal.pone.0153782

**Published:** 2016-04-21

**Authors:** Dena Leshkowitz, Ester Feldmesser, Gilgi Friedlander, Ghil Jona, Elena Ainbinder, Yisrael Parmet, Shirley Horn-Saban

**Affiliations:** 1 Biological Services Department, Weizmann Institute of Science, Rehovot, 76100, Israel; 2 Nancy and Stephen Grand Israel National Center for Personalized Medicine, Weizmann Institute of Science, Rehovot, 76100, Israel; 3 Industrial Engineering and Management Department, Ben-Gurion University of the Negev, Beer Sheva, 84105, Israel; Tel Aviv University, ISRAEL

## Abstract

One of the key applications of next-generation sequencing (NGS) technologies is RNA-Seq for transcriptome genome-wide analysis. Although multiple studies have evaluated and benchmarked RNA-Seq tools dedicated to gene level analysis, few studies have assessed their effectiveness on the transcript-isoform level. Alternative splicing is a naturally occurring phenomenon in eukaryotes, significantly increasing the biodiversity of proteins that can be encoded by the genome. The aim of this study was to assess and compare the ability of the bioinformatics approaches and tools to assemble, quantify and detect differentially expressed transcripts using RNA-Seq data, in a controlled experiment. To this end, *in vitro* synthesized mouse spike-in control transcripts were added to the total RNA of differentiating mouse embryonic bodies, and their expression patterns were measured. This novel approach was used to assess the accuracy of the tools, as established by comparing the observed results versus the results expected of the mouse controlled spiked-in transcripts. We found that detection of differential expression at the gene level is adequate, yet on the transcript-isoform level, all tools tested lacked accuracy and precision.

## Introduction

Ten years ago, short read next-generation sequencing (NGS) technologies first appeared on the market. During the past decade, notable progress has been made in terms of speed, read length, and throughput, along with a sharp reduction in per-base cost. RNA-Seq for transcriptome genome-wide analysis has become one of the most central applications of NGS. With the explosion of analyzed RNA-Seq data sets, it has become apparent that alternative splicing (AS) is a key contributor to cellular diversity in both normal and diseased tissues [1–5]. AS is prevalent in multicellular organisms, affecting approximately 90%–95% of genes in mammals [6]. It can be achieved via exon skipping, intron inclusion, mutually exclusive exons, alternative 5′ or 3′ exon splice sites, alternative promoter usage and alternative polyadenylation site usage. AS enables coding and production of multiple mRNA variants or isoforms from a single gene [4, 6–8]. The resulting isoforms differ in untranslated regions that regulate transcript localization, stability, or translation, or in regions encoding protein-protein interactions or sites for post-translational modification [3]. Overall, AS generates regulatory and functional diversity and complements differential gene expression in biological systems. In addition to quantification of known AS, in some cases it is required to define novel alternatively spliced transcripts. Thus, the ability to accurately build-assemble or quantify and detect differentially spliced transcripts can be of great biological importance.

Multiple bioinformatics tools designed to analyze RNA-Seq on the transcript level, have been developed and reviewed [1, 9, 10]. Although multiple studies have evaluated and benchmarked RNA-Seq tools dedicated to gene level analysis [11–14], few have evaluated its performance on the transcript-isoform level [15]. As pointed out in the reviews referenced above, there is a need for such an evaluation. Angelini et al. [16] concluded that it is difficult to obtain reliable transcript abundance estimates. In a study assessing transcriptome reconstruction method for RNA-Seq, it has been claimed that assembly of complete isoform structures poses a major challenge [17].

To evaluate the performance of the RNA-Seq platform and tools, externally and controlled quantities of transcripts can be added to RNA samples (spike-in). ERCC is a branded set of such RNA standards [18], which consists of 92 polyadenylated bacteria transcripts that mimic natural eukaryotic mRNAs. They are designed to have a wide range of lengths (250–2,000 nucleotides) and GC-contents (5–51%) and can be spiked into RNA samples before library preparation at various concentrations (10^6^-fold range). This set of spike-ins has been used to evaluate reproducibility and to normalize RNA-Seq data [19, 20].

Herein, we have used a novel spike-in approach to evaluate the accuracy of RNA-Seq bioinformatics tools in determining transcript structure and quantifying and detecting differently expressed transcripts. Forty seven mouse transcripts were synthesized and added to mouse RNA samples, allowing for analysis of both the endogenous mouse and the spike-in transcripts with the same methods. To the best of our knowledge this is the first RNA-Seq mammalian study using synthetic spike-in transcripts derived from the same species as the total RNA. The advantage in using the same species spike-ins is that they perfectly mimic the endogenous transcripts in a real biological setting. They contain the exon-intron structure and were designed to contain AS, not existing in bacterial ERCC. This novel approach was used to examine the spike-ins observed versus expected results using a comprehensive set of public and commercial tools representing different bioinformatics approaches to RNA-Seq analysis.

## Materials and Methods

### Cells and RNA

Embryoid bodies (Ebs) were generated by 4 days incubation of single cell suspension (35000cells/ml) of R1 mouse embryonic stem (mES) cells in 5 ml mES growth medium (DMEM, 15% FBS, L-Glutamine, Non-Essential Amino Acids, Pen/Strept, 2-beta–Mercaptoethanol, without Lif) in 6 cm low cell binding dishes. Four-day-old Ebs were treated for 4 days with 2μM RA (Retinoic Acid, Sigma R2625). The day of RA addition corresponds to the sample name day 0. Days 3 and 4 correspond to the samples that had been treated with RA for 3 or 4 days respectively. Day 3 RNA was analyzed only by microarrays. RNA was extracted with an RNeasy Mini kit (Qiagen). RNA sample quality was assessed using TapeStation.

### Microarray data acquisition

Purified RNA from day 0, day 3 and day 4 was reverse-transcribed, amplified, and labeled with Affymetrix GeneChip whole transcript sense target labeling kit. Labeled cDNA was analyzed using Affymetrix Mouse Gene 1.0 ST microarrays, according to the manufacturer's instructions. Microarray data were analyzed using Partek Genomic Suite software. CEL files (containing raw expression measurements) were imported and data was preprocessed and normalized using the Robust Multichip Average (RMA) algorithm. Genes that their intensity was less than 6 and their intensity standard deviation was below 0.3 were considered undetectable and therefore possible candidates for spike-ins.

### Preparation of mouse spike-in transcripts

cDNA clones from the Fantom2 mouse cDNABook collection (RIKEN, Japan) were used as the source for spike-ins. This collection contains a repertoire of splice variants for many mouse genes, and the structure of the collection enables in-vitro transcription of all the cDNAs from either the T7 or T3 promoter.

All the cDNA clones, which were in one of two bacterial plasmid backbones (pFLCI or modified Bluescript) were grown. The plasmid was then purified and quantified, and when possible, the cDNA was fully sequenced from both ends to verify the identity and the nature of the splice variant. For *in-vitro* transcription (IVT) each plasmid was linearized by digesting 2 μg after the poly(A) tail. Next, 500 ng from the linearized plasmids were subjected to IVT for 1 hour using the Ampliscribe T7-Flash transcription kit (Epicentre), according to the manufacturer recommendation. RNA was then precipitated and resuspended in 50μl RNase-free water. The concentration of the produced RNA was measured using a NanoVue (GE Healthcare), brought to 100 ng/μl, and finally run on a TapeStation (Agilent) to verify that the transcript length was accurate before being stored at -80°C.

### Preparation of RNA samples with spike-ins mixes

The 47 IVT-made spikes were combined in mixes. An additional spike was not taken into account as a spike-in or a false positive due to an error in its concentration calculation (gene name: 1700047L14Rik). All spike-in mixes were prepared on the same day. First, the molar concentrations of each transcript and the dilution factors and volumes to be added to each mix were calculated. Next, four serial dilutions (1:10–1:10,000) were prepared for each transcript and the appropriate volumes from the corresponding dilution were combined into each mix. The final composition of each mix is detailed in S1 and S2 Tables. Finally, each mix was thoroughly mixed, aliquoted and stored at -80C.

**Fig 1:**
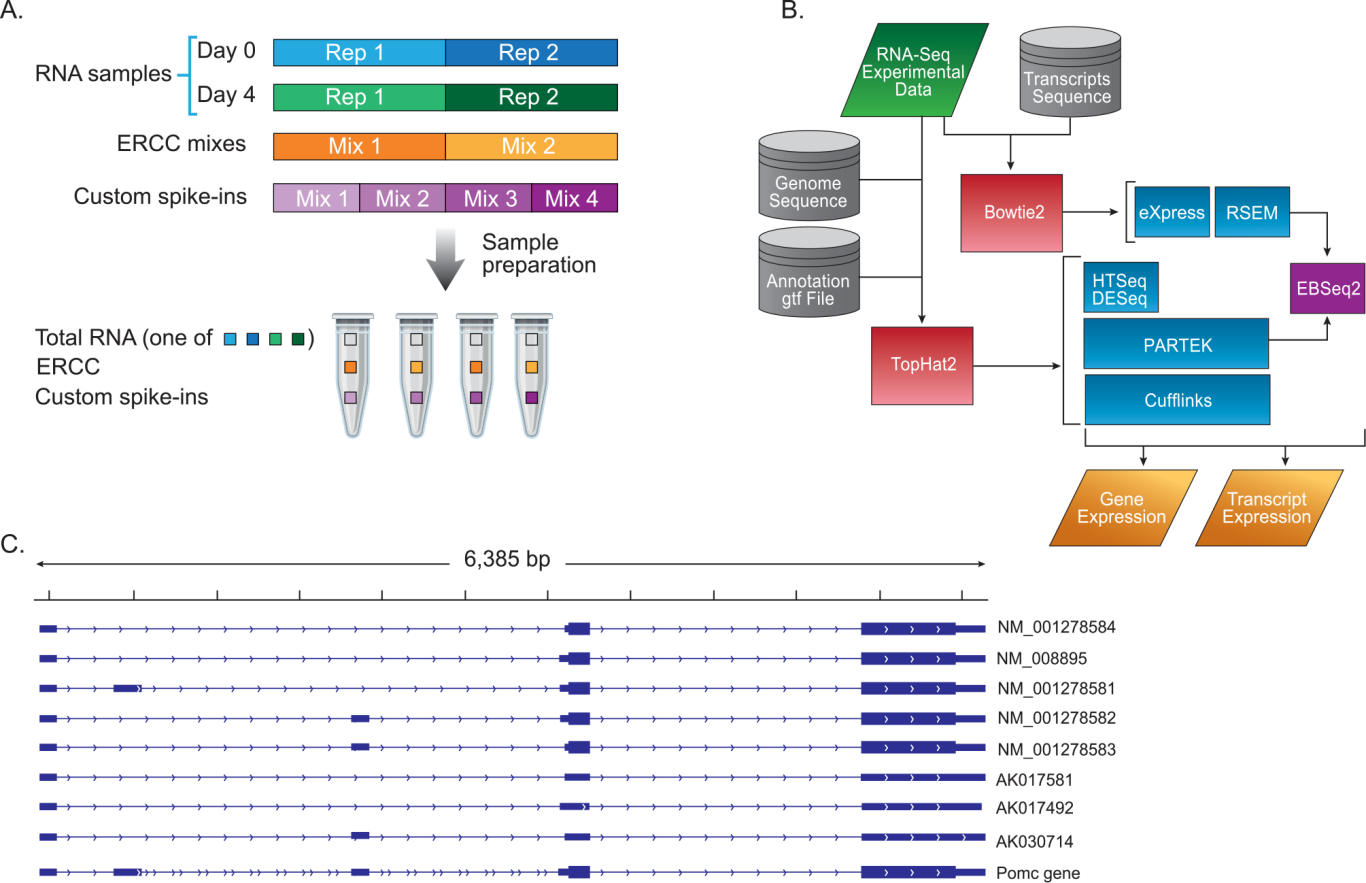
Experiment and analysis design. (A) Illustration of the experimental sample preparation. The experiment consisted of four RNA samples, two replicates (designated as Rep) of Day 0 (colored in blue) and two of Day 4 (green), each sample was divided to four vials, to two of them ERCC mix 1 was added and to other two mix 2 (orange). In addition one of the custom IVT spike-ins was added to each vial (violet). In total there were 16 vials. Custom mix 4 is the control without IVT spike-ins. (B) Diagram of the bioinformatics analysis design. (C) Genome browser view depicting the gene locus of Pomc that includes three distinct spike-in transcripts (AK030714, AK017581 and AK017492).

Mouse IVT spike-in and ERCC mixes were added to the total RNA (S3 Table and Fig 1A). Two microliter of the relevant mix were added to 1 μg of total RNA. The concentrations of all the spike-ins depicted in this study are in accordance to their concentration in the mix.

### Library construction and sequencing

Total RNA (700 ng) (including spike-in mixes) was processed using the Illumina TruSeq Strand Specific total RNA with RiboZero Gold protocol (Illumina). The quality of the libraries was evaluated using Qubit and TapeStation. Sequencing libraries were constructed with barcodes to allow multiplexing of 9 samples on three lanes. Paired-end 100-bp reads were sequenced on Illumina HiSeq Rapid 2500 instrument, using protocols RTA (1.17.21.3) and HCS (2.0.12.0). The sequence yield was between 39–59 million per sample.

### Sequence data analysis

#### Transcriptome mapping and assembly

Above 81% of the reads were aligned to the mm10 genome build and the sequences of the External RNA Control Consortium (ERCC) using TopHat (v2.0.10) [21]. TopHat was run with the option “—library-type fr-firststrand”.

TopHat mapped reads were assembled to transcripts by running Cufflinks [22–24] (2.2.1) (—library-type fr-firststrand). Each sample's mapped reads were assembled by running Cufflinks RABT [23] (reference annotation based transcript assembly; parameter -g) using the gene structures of RefSeq or Gencode (mm10) and additional transcripts used as spike-ins (that were not present in the original gtf) as well as ERCC spikes; this annotation will be termed herein RefSeq+ or Gencode+. Alternatively, Cufflinks was run without any knowledge on transcripts and gene structure (*de novo*). Cuffmerge was then used to compile and join the set of transcripts from each sample, along with RefSeq+ (-g option), thereby creating the gene and transcript structure for the *de novo* and the RABT mode.

In addition, Trinity (v2.0.2) [25] was used to perform *de novo* assembly of the transcripts without reference genome, with the option “—SS_lib_type FR” added to the command. To assess the quality of the built transcriptome, the extent and identity of the overlap between the built transcripts and the spike-ins was evaluated using Blat [26]. The assembly score was calculated by the Transrate software[27].

Unless otherwise specified, all the tools were applied using default parameters.

#### Transcriptome quality control

The rRNA content was measured by using the intersectBed command (bedtools) [28] of the alignment bam files against rRNA and tRNA intervals (the intervals were downloaded from the UCSC tables; four additional intervals that were present in Gencode were added). The number of reads that mapped to these intervals was counted. The percent of reads that mapped to rRNA had a median of 0.17%, with 5.2% being outliers (sample K, see S3 Table).

#### Transcriptome quantification and differential expression analysis

Various tools were used for quantification of the transcripts and genes (Fig 1B). The programs HTSeq [29], Partek Genomic Suite (v6.6; St. Louis, MO) and Cuffdiff [30] were used to quantify the transcripts and genes annotated by RefSeq+ or Gencode+, using TopHat mapped reads as input.

DESeq2 (1.4.5) [31, 32] gene normalization and differential expression analysis were performed after running HTSeq (—stranded = reverse -t exon -m intersection-strict -q -i gene_id). Differential expression of spiked genes within the mixes was analyzed separately for the RNA samples from day 0 and day 4 using DESeq2 parameters (cooksCutoff = TRUE and independentFiltering = TRUE). The normalized counts were converted to FPKM.

Partek® Genomics Suite® software, version 6.6 Copyright; 2014 (St. Louis, MO, USA) with the RNA-Seq workflow was used, and the quantification step, was run with option “yes” for the question “Can assay discriminate between sense and antisense transcripts?” Otherwise, default settings were used. A statistical overview of the mapping results is shown in S4 Table. Four separate ANOVA tests were applied to determine differentially expressed transcripts and genes within the mixes for RNA sampled from either day 0 or day 4. For the ANOVA test, preprocessing consisted of excluding non-expressed gene or transcripts (RPKM = 0), applying an offset of 0.1 and the RPKM values were log2 transformed.

Cuffdiff was used for quantification and differential expression analysis and was run with the RABT gene structure file (parameter—library-type fr-firststrand). In addition Cuffdiff was run using the RefSeq+ or Gencode+ annotation file (without prior run of Cufflinks). The differential expression analysis of samples derived from day 0 and day 4 was performed on two separate runs of Cuffdiff. Plots of quantification data was done using the means of the biological replicates.

The tools eXpress [33] and RSEM (v1.2.18) [34] quantified the transcripts by directly running bowtie (v2.1.0) [35] (Fig 1B) on the transcript sequences (RefSeq+). For eXpress (v1.5.1) (—fr-stranded), the input of bowtie2 run (—no-discordant -X 800 -p 3 -a—norc) was used. For RSEM, first the transcript reference was prepared (rsem-prepare-reference), followed by rsem-calculate-expression (parameter:—bowtie2—forward-prob 0—paired-end). A script provided by the Trinity [25] programmers: abundance_estimates_to_matrix.pl was used to merge the abundance estimation of the different samples and to obtain trimmed mean of M values (TMM) normalized FPKM matrix. Quantification results of HTSeq and RSEM were further normalized by using CQN [36]. ROC curves were calculated using pROC package [37]. Power analysis was performed with Scotty [38] and R package RNASeqPower [39].

The spike-ins plots were done using Matlab, Partek and R. For spike-ins quantification plots an offset of 10e-3 was added to FPKM values before log2 transformation.

EBSeq [40] was used to detect differentially expressed transcripts using the RSEM transcripts quantification output. Fifteen patterns were defined for analysis of samples from the same day.

#### Linear Regression Model

Linear regression models were used to explain the observed amount of spike-ins (FPKM values). The model included the basic explanatory attributes: expected spike-in amount, day of sample (0 or 4) and replicate sample information. In addition to the basic attributes, the spike-in characteristics %GC and the length (number of bases in linear and squared centralized terms) were included. The initial model consisted of the basic effects and all second-order interactions effect, excluding the sample replication information. The final basic model was achieved after implementation of the backward elimination algorithm using the step function in R (3.2). To achieve a better model fit, the log of the observed and expected amounts were used. In order to test if the spike-in characteristics significantly improved the fit, this model procedure was run twice, once with only the basic attributes and once with all attributes. The third model included in addition to the attributes described above, the loci attributes of the spike-ins: the number of alternative spliced isoforms within the gene locus and the number of alternative spike-ins added per locus. The confidence intervals for the adjusted R-squared were calculated using the MBESS package in R [41].

## Results and Discussion

### Study design

This study focuses on the expression analysis at the transcript level. Furthermore, this is the first study to perform an evaluation of bioinformatics tools based on mouse transcripts spike-ins added to mouse RNA samples. Our design of mouse spike-ins included splice variants from the same genetic background, tested over a wide range of concentrations.

Mouse total RNA was extracted from EBs before (designated as day 0) and four days after the addition of RA (designated as day 4). RA plays multiple roles in the nervous system, including induction of neural differentiation, axon outgrowth and neural patterning [42]. This procedure was repeated to yield two biological replicates (Fig 1A). Mouse spike-ins of 47 different mouse RNA transcripts, generated in vitro from plasmid constructs (see Methods), were added to the total RNA samples. These transcripts were selected as their expression levels were below the detection threshold, as determined by prior microarray analysis (results not shown). 23 of the spike-ins originated from 10 different genomic loci (S1 Table); namely, each locus was represented by at least two different isoform-transcripts termed in this study, as multi-spike-ins loci. The remaining 24 spike-ins represented different loci (S2 Table) and were termed single spike-in loci. The selection criteria for the transcripts used as single spike-in loci, were undetectable expression levels and transcripts with a diverse length and GC content. Three different mixes of spike-ins were prepared, each containing combinations of all the spike-ins, at 10-fold increments, for example AK008207 is found at 1000, 10 and 100 attomoles/μl in mix 1, mix 2 and mix 3 respectively (S1 and S2 Tables). A control mix containing only native total RNA was added (mix 4). Three of the spike-ins were added at low concentrations (0.1–100 attomoles/μl) and three were added at high concentrations (100–10000 attomoles/μl). Each of the four total RNA samples (duplicates of day 0 and day 4) was divided into four tubes and the mixes were then added to each. In addition, the ERCC spike-ins were added as described (S3 Table, Fig 1A). Due to the large fold changes between the spike-ins amounts in the mixes (10–100), we choose to work with duplicates, see power analysis (S1 and S2 Files). The replication quality was assessed using the transcript (RSEM) and gene expression levels (HTSeq and DESeq2) on the control samples (mix 4). Pearson correlations between the duplicates on the transcript level were 0.88 (day 0) and 0.96 (day 4) and on the gene level were 0.95 (day 0) and 0.99 (day 4). The RNA was sequenced as described in the Methods section.

### Quantification and Differential Expression of Spike-ins

In order to assess and compare the ability of the various bioinformatics approaches and tools to quantify transcript levels, we analyzed the Illumina reads with different tools (Fig 1B). These tools differ in the initial mapping stage requirement: RSEM and eXpress use the outputs produced from mapping the reads to transcript sequences, whereas the other tools used. i.e., Cufflinks, Cuffdiff, DESeq2 and Partek, use the output produced after mapping the reads to the genome.

All the methods applied, except for DESeq2, support transcript level analysis and utilize the expectation–maximization (EM) algorithm in order to distribute the reads amongst the transcripts. Yet, they differ in handling multi-mapping events and sequence-bias [22, 24, 30, 33, 34, 43].

**Fig 2:**
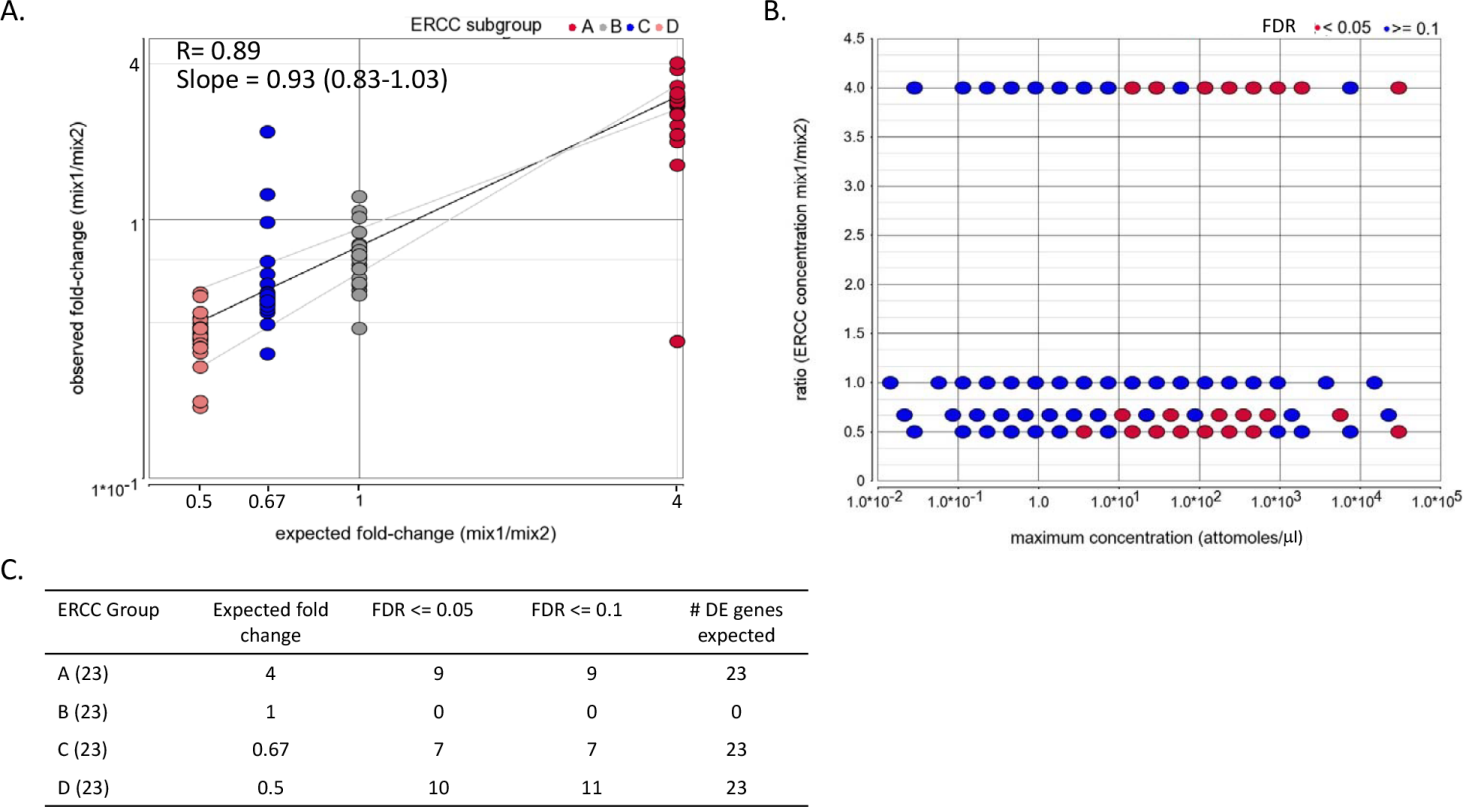
Analysis of ERCC spike-ins. Differential expression of ERCC spike-ins. (A) Scatter plot of DESeq2 normalized ratio between ERCC mix2 and ERCC mix 1 versus the expected ratio for day 0. Note the axes scale in the plot is logarithmic. The correlation coefficient and the slope were calculated on the log values. The gray lines represent 95% confidence interval of the slope. The slope confidence interval is indicated in brackets. (B) Scatter plot illustrating the relationship between the spike-in levels and differential expression detection capacities. The fold changes between the two ERCC mixes and the FDR were calculated using DESeq2. The maximum concentration (log_10_) within the pairwise comparison is shown in the X- axis. (C) Number of significantly differentially expressed (DE) ERCC spikes.

**Fig 3:**
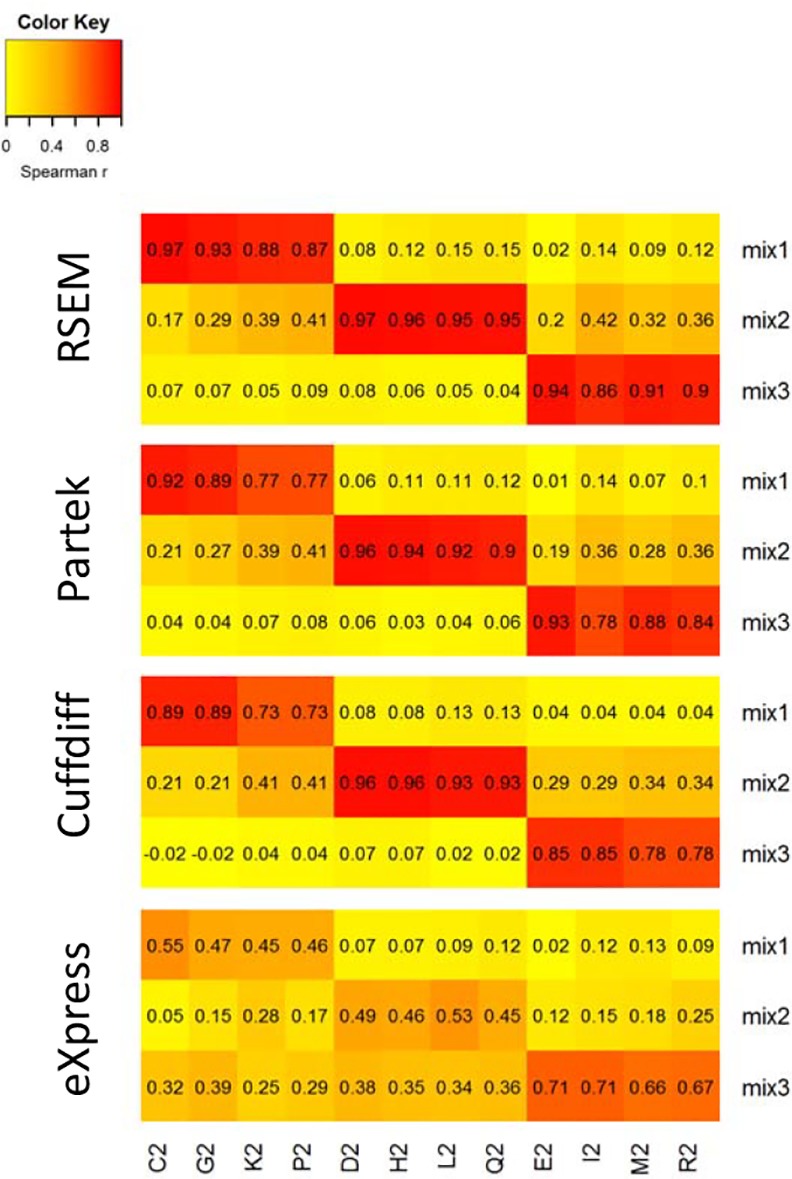
Spike-in correlations between expected versus observed values. Spike-ins Spearman correlation between concentration values (mix 1, mix 2, mix 3) and observed FPKM values. Sample names are shown in the figure and described in the S3 Table.

**Fig 4:**
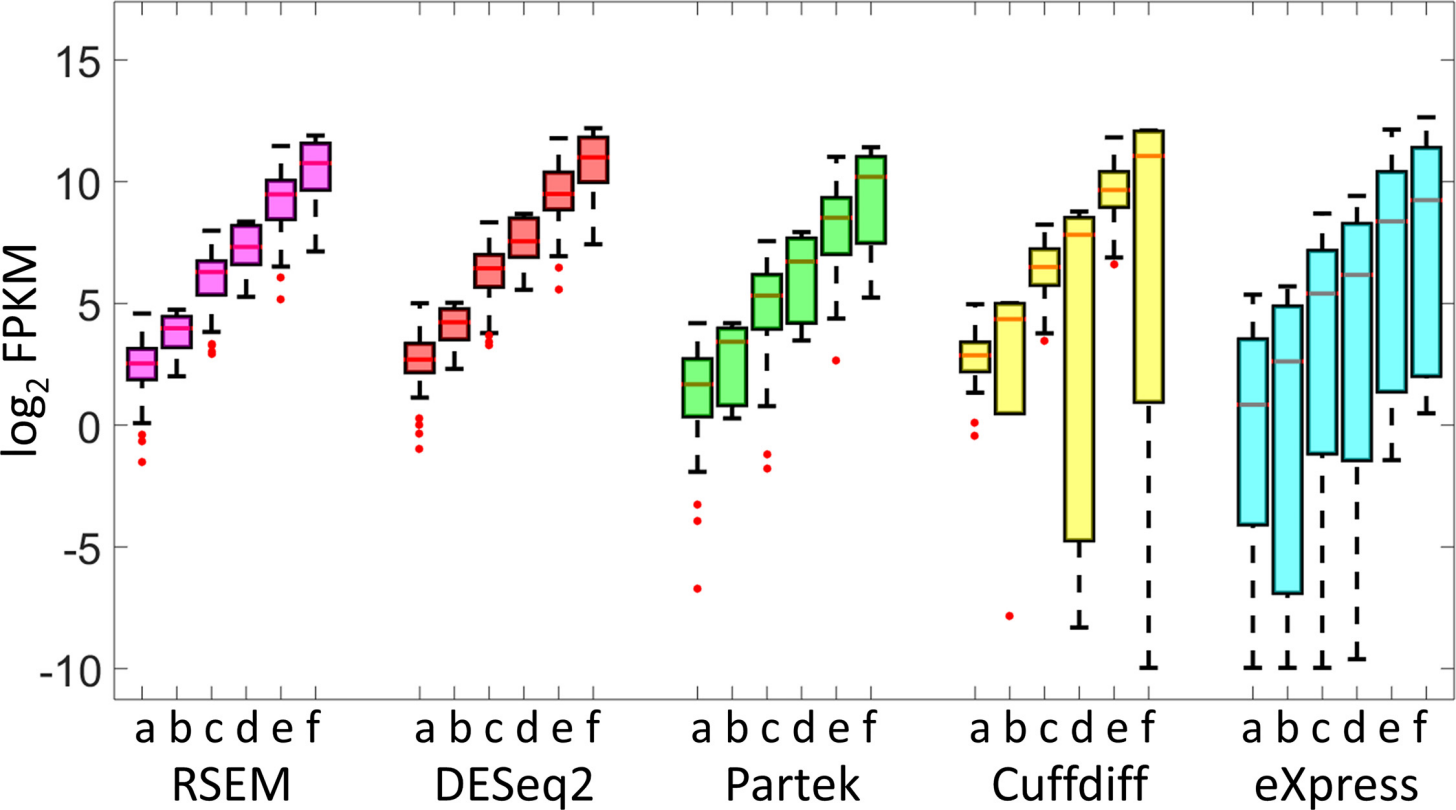
Abundance estimation of single locus transcript spike-ins using various bioinformatics tools. Spike-ins were quantified using various tools: RSEM, Partek, DESeq2, Cuffdiff and eXpress. Depicted are box plots of the observed versus the expected values for 19 spike-ins. In the X-axis (a-f) depicts spike-ins at a concentration of 50, 100, 500, 1000, 5000 and 10000 attomoles/μl respectively. The boxplots for the concentrations 50, 500 and 5000 attomoles/μl are based on 64 observations, the other three box plots are based on 12 observations.

**Fig 5:**
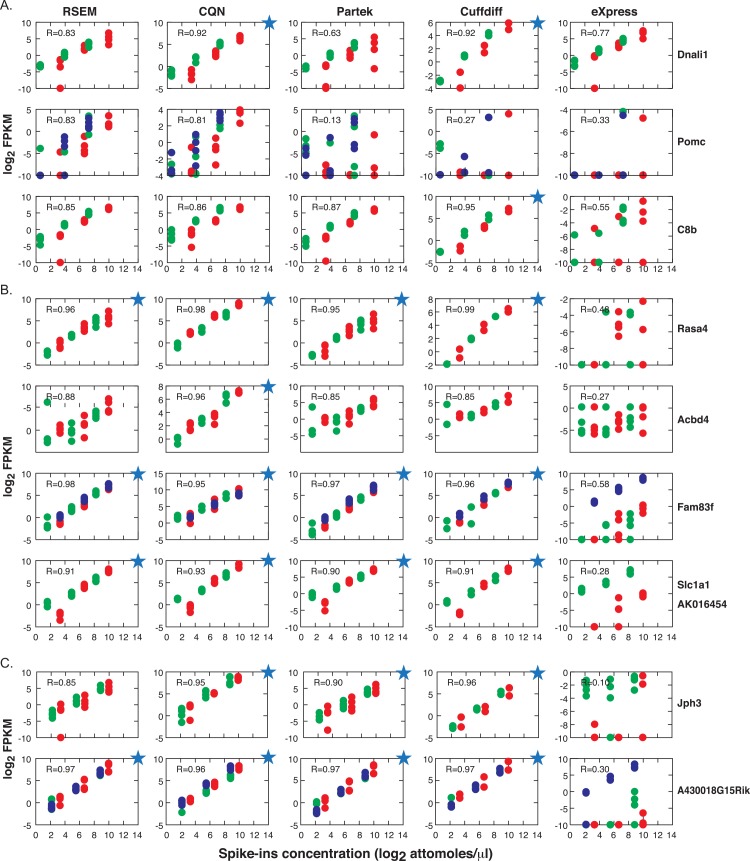
Abundance estimation of spikes with multiple isoforms per locus, using various bioinformatics tools. Spike-ins were quantified using various tools: RSEM, RSEM normalized by CQN, Partek, Cuffdiff and eXpress. Depicted are scatter plots of the observed versus the expected values, for the spike-ins that have multiple isoforms per locus. The loci are arranged by the concentration of the minor transcript: 15% (A), 30% (B), 45% (C). The major transcripts are depicted as red points and the minor transcripts in green and blue. Blue stars depict plots with a correlation of > = 0.9.

**Fig 6:**
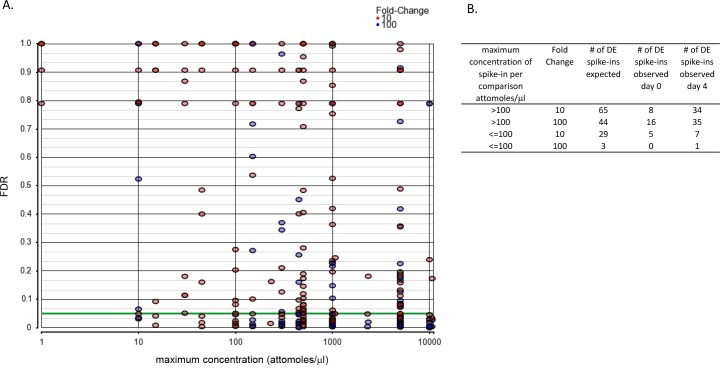
The relationship between the spike-in concentration and differential expression detection capacities. A scatter plot displaying association between spike-in concentrations and the ability to detect differential expression. Shown are the Partek analysis of day 0 and day 4 samples for the three mixes (1–3). X axis: the maximum spike-in concentration within the pairwise comparison. The color intensity is proportional to the number of points at each coordinate. The green line indicates the FDR < = 0.05 cutoff.

Quantification of transcripts is greatly influenced by the number of potential isoforms and by incorrect or misassembled isoforms that can introduce uncertainty. Therefore, all quantification results shown here (Figs 2–6) were obtained using the same set of transcripts, namely, the gene structures of RefSeq+ (see definition in Methods) without novel assembled transcripts.

#### Analysis of ERCC spike-ins

After TopHat mapping to the mm10 genome, RefSeq+ genes were quantified by HTSeq and normalized expression values were extracted with DESeq2. Between 0.25–0.79 million reads uniquely aligned to ERCC spike-ins and were efficiently detected, as shown by the relationship between the ERCC spike-in counts (DESeq2 rld values) and their nominal concentration (S1 Fig). Rld stands for regularized log transformation of the original count data to a log2 scale by fitting a model with a term for each sample and a prior distribution on the coefficients which is estimated from the data. Furthermore, the relationship between the observed and expected ratio (ERCC mix 1 over ERCC mix 2 for the same RNA sample) had a linear correlation coefficient (R) of 0.89 in day 0 (Fig 2A) and 0.85 in day 4 (S2 Fig).

DESeq2 was used to detect differentially expressed ERCC genes by comparing the eight samples which had ERCC mix 1 versus the eight samples which had ERCC mix 2 (Fig 2B). In this comparison, only the ERCC genes should be detected as differentially expressed (since the comparison sets are balanced as far as the day and IVT mixes). Only 26 of the 69 spikes were detected with false discovery rate (FDR) less or equal to 0.05 (38%). ERCC spike-ins below a concentration of 1 attomoles/μl were not detected as differentially expressed (Fig 2B). The differentially expressed spike-in genes are not necessarily the ones with the highest fold changes (see ERCC groups in Fig 2C), implying that in addition to the maximum concentration of the spike-in transcripts and the fold change, other specific factors, such as sequence and length, influence detection of differential expression.

#### Analysis of IVT mouse spike-in quantification

After mapping to either the genome or the transcripts, transcripts were quantified by the various tools shown in Fig 1B. The selected tools differ in the methodology implemented for detection of differentially expressed genes. DESeq2 and Cuffdiff model the count variance across replicates as a nonlinear function of the mean counts and use a Negative Binomial distribution to model biological variability, and provide a measure of statistical significance in the absence of a large number of biological replicates [13]. In contrast, Partek uses an ANOVA model. EBSeq [40] takes advantage of the merits of empirical Bayesian methods.

Quantification of the spike-ins in mix4 samples corroborated the undetectable endogenous level of expression of these transcripts, with a few exceptional transcripts with up to 2 FPKM. The correlation between the observed FPKM values of the 47 IVT spike-ins and the nominal IVT spikes-in concentrations is depicted in a heat map plot (Fig 3). There were four samples for each mix and, as expected, the highest pair correlations were within the same mix. The correlation values were highest when using the RSEM tool (maximum correlation value 0.97) and lowest when using eXpress (maximum correlation value 0.71). CQN normalization [36] was applied on the RSEM counts, taking into account the transcripts GC content and length. The spike-in correlations between the expected and the CQN values had the same values as RSEM with TMM normalization.

Analysis of the distribution of the FPKM values for the single spike-in loci (Fig 4 and S3 Fig), showed that quantifications by RSEM, DESeq2 (gene level analysis) and Partek provided a narrow distribution of values, whereas the quantification by Cuffdiff and eXpress yielded a broad distribution of values for spike-ins added at the same nominal concentration. Some transcripts were categorized as having low expression even though they were added at significant concentrations (>100 attomoles/μl), for example Wnt7b in the Cuffdiff analysis (S3 Fig).

In the analysis of the expression level of the 10 multi-spike-ins loci (Fig 5, S4 Fig), the performance of RSEM, RSEM followed by CQN, Partek and Cuffdiff was closer to the predicted behavior, when compared to eXpress. The latter provided the most scattered values. Interestingly, the ability to quantify the concentration of the minor isoform spike-in within a locus, did not differ dramatically among the three groups (10%, 30% or 45% of the major transcript in the loci).

#### Analysis of IVT spike-ins differential expression

One of the most important applications of RNA-Seq is the study of differential expression of gene and transcript between two or more biological conditions. Table 1 shows the expected number of differentially expressed transcripts between RNA samples of different mixes (day 0 mix 1 versus day 0 mix 2, etc.) and the results observed with the various analysis tools. Note that when comparing the biological replicates of the same day, the expected differentially expressed transcripts should only be the IVT spike-ins. For each comparison, differentially expressed transcripts were defined as those with a FDR value < = 0.05. Theoretically, there should be 564 such values (47 spike-ins multiplied by 12 comparisons).

**Table 1:** Number of differentially expressed transcripts among the mixes, as identified using various bioinformatics tools and RefSeq+ annotation.

	PARTEK		Cuffdiff		Cuffdiff (with de novo)*		EBSeq with RSEM counts	
Description	all	spikes	all	spikes	all	spikes	all	spikes
mix 1 vs. mix 2- Day 0	48	10	0	0	0	0	775 (41)	39 (27)
mix 1 vs. mix 3- Day 0	52	10	0	0	0	0		
mix 1 vs. mix 4- Day 0	63	19	0	0	0	0		
mix 2 vs. mix 3- Day 0	11	9	0	0	0	0		
mix 2 vs. mix 4- Day 0	29	17	0	0	0	0		
mix 3 vs. mix 4- Day 0	10	7	0	0	0	0		
mix 1 vs. mix 2- Day 4	90	24	33	29	47	3	932 (27)	44 (29)
mix 1 vs. mix 3- Day 4	103	29	35	29	50	3		
mix 1 vs. mix 4- Day 4	96	25	20	13	17	0		
mix 2 vs. mix 3- Day 4	79	24	30	28	42	2		
mix 2 vs. mix 4- Day 4	80	29	17	13	19	0		
mix 3 vs. mix 4- Day 4	59	25	14	9	12	0		
**Sum**	**720**	**228**	**149**	**121**	**187**	**8**	**1707**	**83**
**expected**	** **	**564**	** **	**564**	** **	**564**	** **	**94**
**% false positive**	** **	**68.33**	** **	**18.79**	** **	**95.72**	** **	**95.14**
**% identified**	** **	**40.43**	** **	**21.45**	** **	**1.42**	** **	**88.30**

Bioinformatics tools were applied to determine differentially expressed transcripts (FDR< = 0.05),”all” depicts all the differentially expressed transcripts. First number in EBSeq analysis is the number of transcripts which were not pattern 1, in brackets is the number of transcripts that were pattern 15.

*Transcripts of class “=“, “j” or “x”

Using Partek, only 40.4% of the IVT spike-ins comparisons were identified as statistically significant (FDR< = 0.05), and 68.3% of the significant comparisons were false-positives. Cuffdiff identified 21.4% of the comparisons of the IVT spike-ins as significant and had false-positive rate of 18.8%. In addition, we ran Cuffdiff with the transcripts definition created by Cufflinks, which allowed for *de novo* transcript assembly (option rabt in the software). This annotation file had many more transcripts than the RefSeq+ file (107,339 versus 33,286) and was less efficient in detecting differentially expressed spike-in transcripts. Fewer significant comparisons for the IVT spike-ins (1.4%) and a dramatic increase in false detection rate (95.7%) were observed. When using Gencode+ annotation (contains 136078 transcripts; see Table 2), no transcripts were detected as differentially expressed with Cuffdiff, whereas with Partek a similar amount was spike-ins were detected (38.3%), yet there was a dramatic increase in false positive detection (91%). Hence, an increase in the number of annotated transcripts causes a decrease in the ability to reliably detect differentially expressed transcripts.

**Table 2:** Number of differentially expressed transcripts and genes among the mixes, as identified using various bioinformatics tools using Gencode+ annotation.

	Transcripts				Genes					
	Partek		Cuffdiff		Partek		Cuffdiff		DESeq2	
Description	all	spikes	all	spikes	all	spikes	all	spikes	all	spikes
mix 1 vs. mix 2- Day 0	187	12	0	0	56	12	25	25	27	26
mix 1 vs. mix 3- Day 0	156	11	0	0	53	10	18	18	29	27
mix 1 vs. mix 4- Day 0	174	19	0	0	54	16	11	11	23	22
mix 2 vs. mix 3- Day 0	64	11	0	0	10	8	19	19	30	28
mix 2 vs. mix 4- Day 0	81	17	0	0	17	12	11	10	20	19
mix 3 vs. mix 4- Day 0	30	6	0	0	9	8	12	11	21	19
mix 1 vs. mix 2- Day 4	273	19	0	0	112	25	33	30	36	32
mix 1 vs. mix 3- Day 4	319	26	0	0	122	26	31	28	36	32
mix 1 vs. mix 4- Day 4	330	23	0	0	131	27	21	17	29	26
mix 2 vs. mix 3- Day 4	155	19	0	0	110	21	30	27	36	31
mix 2 vs. mix 4- Day 4	305	28	0	0	113	25	20	17	27	24
mix 3 vs. mix 4- Day 4	320	25	0	0	122	23	17	14	24	21
**Sum**	**2394**	**216**	**0**	**0**	**909**	**213**	**248**	**227**	**338**	**307**
**Expected**	** **	**564**		**564**	** **	**432**	** **	**432**	** **	**432**
**% false positive**	** **	**90.98**		**0.00**	** **	**76.57**	** **	**8.47**	** **	**9.17**
**% identified**	** **	**38.30**	** **	**0.00**	** **	**49.31**	** **	**52.55**	** **	**71.06**

Bioinformatics tools were applied to determine differentially expressed transcripts or genes (FDR< = 0.05),”all” depicts all the differentially expressed transcripts or genes.

To measure the sensitivity and specificity in detecting differentially expressed IVT spike-in controls by the various methods we performed a ROC analysis (S5 Fig). ROC analysis was done by using different cut-off points of the p values, revealing that Cuffdiff (run with RefSeq+ annotation file) method performed better than Partek in detecting the differentially expressed spike-ins, with average (day 0 and day4) area under the curve (AUC) 0.9 for Cuffdiff and an average of 0.6 for Partek (S5 Fig). The ROC analysis was in disagreement with the FDR results, indicating a possible problem in Cuffdiff FDR calculation.

EBSeq, an empirical Bayesian approach, was also tested. For isoform level inference, EBSeq directly accommodates isoform expression estimation uncertainty by modeling the differential variability observed in distinct groups of isoforms. In this study, there were four types of mixes and therefore, 15 possible expression patterns. The IVT spike-ins should be determined as pattern 15: “1234”, since this depicts the situation of a different expression level in each of the mixes. A non-differentially expressed transcript is one which has pattern 1: “1111” i.e., the same level of expression in all the mixes. EBSeq was run on transcript raw counts (non-normalized) derived from RSEM and the number of transcripts that were not pattern 1 (Table 1) as well as those that were explicitly pattern 15 were counted. Although 88.3% of the IVT spike-ins were identified as pattern 15, in total, 1707 transcripts were not categorized as pattern 1, thus the false-positive discovery rate was extremely high 95.1%.

To determine if the differential expression detection of the IVT spike-ins was influenced by concentrations and/or fold differences, the maximum nominal value within a comparison was plotted against the FDR value (Fig 6). The plot shows Partek analysis and demonstrates that the spike-ins were more likely to be detected as differentially expressed if the maximum concentration was above 100 attomoles/μl. However, several transcripts did not follow this trend, such as AK089819 (loci: Irf4_plus) and AK030714 (loci: Pomc_plus).

The above assessment at the transcript level revealed that none of the tools were adequately accurate. The accuracy of the same data at the gene level was evaluated. The spike-ins in each locus were designed to have 10 fold increments in concentration between the mixes. Therefore, differential expression of the spike-in loci (gene level) was expected to be detected.

Analysis of differential expression among the mixes at the gene level, using DESeq2 (this tool does not support transcript analysis, Table 3), revealed that 68.9% of the comparisons of IVT spike-ins loci were identified as differentially expressed and the false discovery rate was close to the expected (observed 7.3%, expected 5%). In comparison, the Partek analysis at the gene level, detected 44.% of the IVT spike-ins loci contrasts between the mixes as differentially expressed, where 27.1% of the differentially expressed gene comparisons were false-positives.The Cuffdiff analysis categorized 55.2% of the spike-in loci comparisons as differentially expressed and 6.8% of the differentially expressed gene comparisons were false-positives. Interestingly, the ability to detect differentially expressed genes was not changed when using Gencode versus RefSeq annotation for both Cuffdiff and DESeq2 whereas with Partek false positive detection increased (from 27.1% to 76.6%; Table 2 and Table 3). It should be noted that in all Cuffdiff analysis described, no spike-ins transcripts were detected as differentially expressed in day 0, yet at the gene level close to half of the spike-ins are identified as differentially expressed.

**Table 3:** Number of differentially expressed genes among the mixes, as identified using various bioinformatics tools using RefSeq+ annotation.

	Partek		Cuffdiff		DESeq2	
Description	all	spikes	all	spikes	all	spikes
mix 1 vs. mix 2- Day 0	33	11	26	26	28	27
mix 1 vs. mix 3- Day 0	33	9	17	17	27	26
mix 1 vs. mix 4- Day 0	37	16	13	13	22	21
mix 2 vs. mix 3- Day 0	9	8	20	20	29	28
mix 2 vs. mix 4- Day 0	17	14	14	14	20	19
mix 3 vs. mix 4- Day 0	12	9	13	13	21	20
mix 1 vs. mix 2- Day 4	22	22	32	30	35	32
mix 1 vs. mix 3- Day 4	22	22	34	30	36	33
mix 1 vs. mix 4- Day 4	26	26	25	18	29	26
mix 2 vs. mix 3- Day 4	15	15	31	30	33	30
mix 2 vs. mix 4- Day 4	25	25	18	17	26	23
mix 3 vs. mix 4- Day 4	22	22	20	17	24	21
**Sum**	**273**	**199**	**263**	**245**	**330**	**306**
**Expected**		**444**	** **	**444**	** **	**444**
**% false positive**		**27.11**	** **	**6.84**	** **	**7.27**
**% identified**	** **	**44.82**	** **	**55.18**	** **	**68.92**

Bioinformatics tools were applied to determine differentially expressed genes (FDR< = 0.05),”all” depicts all the differentially expressed genes.

### Building a Linear Regression Model to Explain Spike-ins Quantification Results

Linear regression models were used to explain the observed expression level of single spike-ins quantified by HTSeq and normalized by DESeq2. A comparison of a model designed with both basic explanatory attributes (expected spike-in concentration, sampling day and number of replicates) and spike-in attributes (%GC and length) to a model considering only the basic components, demonstrated a significant contribution of the spike-in attributes (F(6,254) = 18, p< = 0.00001). The final model included the main effect of the expected concentration (log scale), %GC, length (linear and centralized square) and the interaction %GC with all the other factors (Table 4). The final model's adjusted R^2^ square was 0.91 (CI: 0.89–0.92), whereas the model with only the basic attributes had a R^2^ of 0.87 (CI: 0.85–0.89) thus the effect size was minor (S6 Table). We then explored the dependence between expected and observed values using these final models (S6A Fig). In this figure, there are three %GC quantiles (0.25, 0.5, 0.75), and three quantiles for length (0.25, 0.5, 0.75). The intercept in these figures was dependent on the combination of %GC and length.

**Table 4:** Linear regression model using normalized DESeq2-HTSeq FPKM values for single loci spike-ins.

	Estimate	Std. Error	t value	p value
(Intercept)	-8.03	1.99	-4.03	0.00007
expected	1.69	0.15	11.05	0
day4	5.36	0.93	5.75	0
%GC	0.14	0.04	3.44	0.00069
length/1000	1.37	0.57	2.43	0.016
length_sq/1e+06	-2.39	0.68	-3.54	0.00048
expected:%GC	-0.02	0.00	-5.75	0
day4:%GC	-0.10	0.02	-5.23	0
%GC:(length/1000)	-0.03	0.01	-2.59	0.01009
%GC:(length_sq/1e+06)	0.05	0.01	3.68	0.00029

RMSE 1.0

Adjusted R-squared: 0.91

A similar analysis was performed for the observed quantification results of HTSeq normalized by CQN (S5A Table, S6B Fig). The CQN quantile normalization algorithm combines robust generalized regression to remove systematic bias. In our analysis, it was used to remove bias resulting from %GC and transcript length. The final CQN model achieved an R^2^ value of 0.90 (CI: 0.87–0.91), similar to the results of the DESeq2-HTSeq model.

The complete model that explains all the 47 spike-ins using the normalized RSEM FPKM values (Table 5) had, in addition to the attributes described above, the loci attributes. The R^2^ of the complete model was 0.94 (CI: 0.93–0.94), the R^2^ of the model without the loci attributes, was 0.92 (CI: 0.91–0.93). This R^2^ difference was significant (F (6, 547) = 23.36, p< = 0.00001), but the effect size was minor. The model with the basic attributes had an R^2^ of 0.85 (CI: 0.83–0.86). Therefore, we can see that the spike-ins attributes contribute more than the loci attributes to the model. Similar results were obtained with the CQN normalized RSEM values (S5 Table, S5 Fig).

**Table 5:** Linear regression model using normalized RSEM FPKM values for all spike-ins.

	Estimate	Std. Error	t value	p value
(Intercept)	-7.48	1.08	-6.91	0
replica2	-0.37	0.07	-5.32	0
expected	1.69	0.10	17.71	0
day4	5.19	0.56	9.36	0
%GC	0.11	0.02	5.30	0
length/1000	1.77	0.37	4.74	0
length_sq/1e+06	-2.08	0.41	-5.03	0
spiked_loci	0.26	0.12	2.13	0.03371
isoforms_loci	-1.92	0.45	-4.28	0.00002
expected:%GC	-0.02	0.00	-8.54	0
expected:(length/1000)	0.04	0.01	2.94	0.00341
expected:spiked_loci	-0.05	0.02	-2.81	0.00522
expected:isoforms_loci	-0.14	0.04	-3.79	0.00017
day4:%GC	-0.10	0.01	-8.97	0
%GC:(length/1000)	-0.04	0.01	-5.34	0
%GC:(length_sq/1e+06)	0.04	0.01	4.62	0
(length/1000):isoforms_loci	-7.48	1.08	-6.91	0

RMSE 0.82

Adjusted R-squared: 0.94

### Accuracy of Transcriptome Assembly

#### Transcriptome assembly using Cufflinks

**Fig 7:**
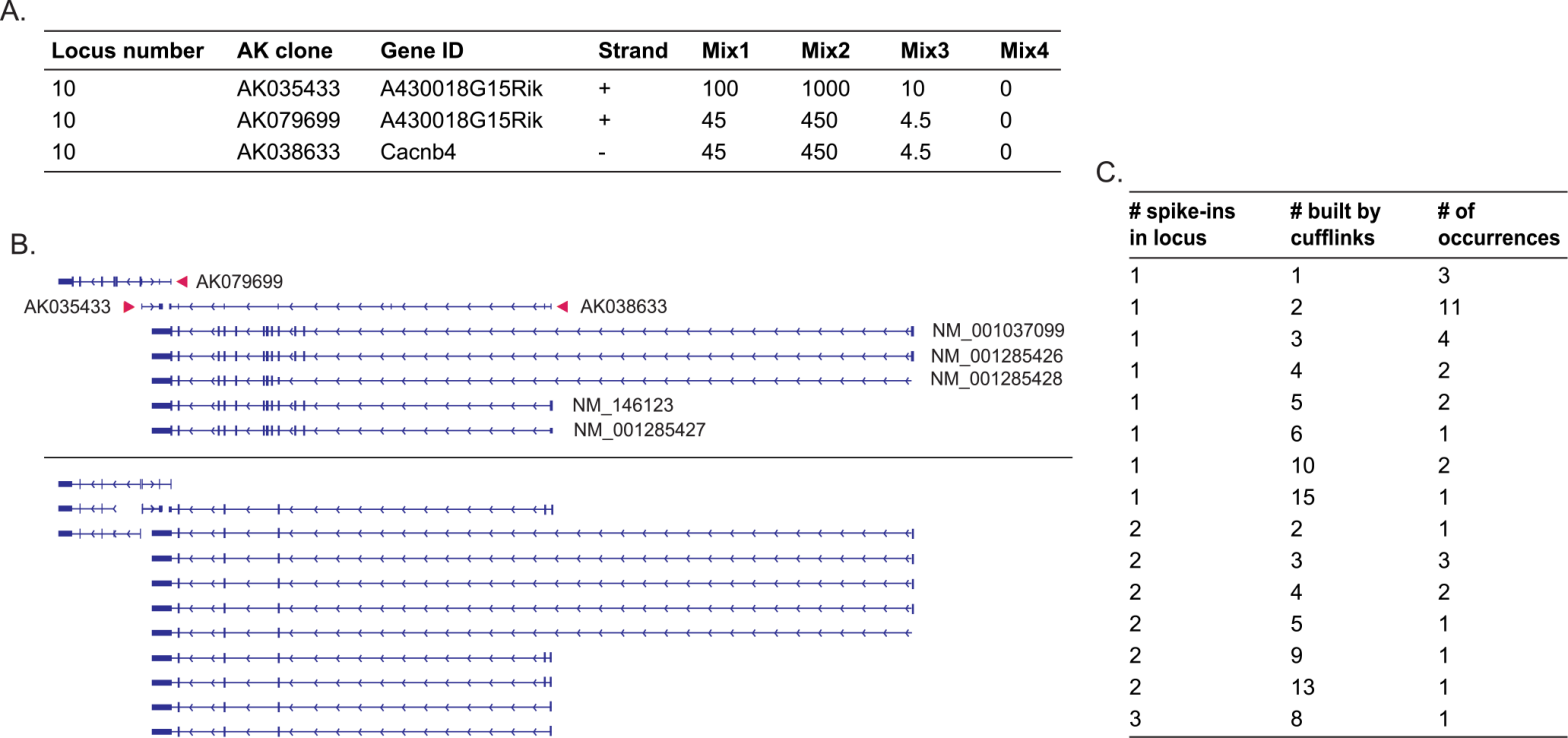
**Cufflinks assembly of transcripts**(A) Information regarding the spike-ins used for locus 10. The concentration of the spike-ins in each mix is shown (attomoles/μl). (B) The RefSeq+ and Cufflinks assembled transcripts structure within locus 10. (C) Table showing the number of transcripts spiked-in and the number of transcripts reported in the Cufflinks assembly output.

Cufflinks [22, 24] was run, without supplying a reference annotation file, in order to evaluate the program's ability to properly assemble the spike-ins. Transcriptomes were separately assembled for each sample and merged to a master transcriptome using Cuffmerge. A total of 40,305 genes and 107,388 transcripts were assembled. All 47 spike-ins were built correctly (class code “=“); in addition, 150 transcripts corresponding to these spike-ins loci were built (class codes “j”, “o” and “x”, identified as a spike-in transcript by the name designated as “nearest_ref” or “old”) (Fig 7C). An example of an assembly of transcripts corresponding to loci 10 is shown in Fig 7A and 7B.

#### Transcriptome assembly using Trinity

Trinity was run using all the sample reads without a reference genome, to build *de-novo* transcripts. A total of 1,220,442 transcripts, belonging to 1,028,937 loci according to the tool definition, were built. The number of overlaps between the transcripts and 47spike-ins was checked. A total of 1056 transcripts displayed at least 90% overlap with at least 90% identity to 45 spike-ins genomic regions. There were 2 single loci spike-ins (not the ones with the lowest concentration), for which none of the Trinity built transcripts met the overlap requirement. To assess the built of the spike-ins, overlap between the spike-ins and the built transcripts was evaluated using Blat. Blat reciprocal overlaps of at least 90% of the sequence and at least 90% identity were found for 34 transcripts. In three additional cases the transcript built by Trinity included the spike-in totally but was approximately twice longer than the spike-in. All the additional 10 spike-ins were partially build, for one of them 35% of the sequence was built, for four of them between 50% and 70% and for five additional ones between 71% and 87% of the original length was built. All these built sequences had and identity of at least 97% to the spike-ins.

A general evaluation of the *de novo* transcriptomes was performed using Transrate. A transrate assembly score is calculated as the geometric mean of all contig scores multiplied by the proportion of input reads that provide positive support for the assembly. The transrate assembly scores were 0.2072 and 0.2069 when using RefSeq+ or Gencode+ annotations respectively.

## Conclusions

The goal of this study was to assess the ability of various bioinformatics tools to assemble, quantify and detect differentially expressed transcripts. The novelty of the presented approach, lay in the utilization of spike-ins of known concentration, from the same organism as the RNA sample, allowing for simultaneous analysis of the spike-in and all biological RNA-Seq transcripts. To date, there has been a lack of transcript level assessment studies that compared analysis tools. The reason for this is the lack of standard transcripts with known concentration, leading to an inability to grade the tool accuracy. This study was performed in samples from two biological developmental stages, testing the tool performance accuracy in different biological settings and evaluating sample bias. Although the experiment was designed with biological duplicates per developmental stage, it had sufficient power to detect the spike-ins fold changes of 10–100. We suggest this spike-ins approach, even though it is time consuming and costly as a dedicated way to evaluate the accuracy of bioinformatics tools, to learn their strength and weakness. Due to the extensive work involved in this tailored approach it is not suitable as a mainstream procedure.

Our results demonstrated variability in the ability of the different tools to properly quantify the mouse spike-in transcripts. We found that the program which does not attempt to remove sequence biases performed better (RSEM). The minimum concentration required to detect accurately a transcript with of 50 million reads per sample was 100 attomoles/μl. The minor isoforms-transcripts within the multiple spike-ins loci could be quantified properly. Furthermore, using linear regression models, we have found that GC content, length and the number of transcript-isoforms per loci had a small effect on the accuracy of RSEM quantification. The RNA background which is different between day 0 and day 4 samples, influenced the spike-ins quantification and differential expression results.

We also found that detection of differential expression at the gene level was adequate when using DESeq2, yet on the transcript-isoform level, all tools tested for differential expression lacked accuracy and/or precision. This result is disappointing given the high fold changes (10–100) used for the spike-ins, while in many biological settings the fold changes are lower. Cuffdiff analysis permitting novel transcripts to be defined, revealed that the ability to determine accurately differentially expressed transcripts decreased as the number of defined transcripts increased. In addition when using a more extended annotation database than RefSeq (Gencode), the differential expression detection of transcripts was less accurate.

Cufflinks-mediated transcript *de novo* assembly with the mouse reference genome, assembled the spike-ins correctly. Yet, many variations to the spike-in transcript isoforms were also assembled. Trinity assembly produced a huge number of transcripts, more than one million, which may have resulted from the vast amount of sequence data used for the Trinity assembly.

To conclude, all the tools tested for identification of differentially expressed isoforms-transcripts and for de-novo assembly of RNA-Seq short reads, were found to be insufficient and required improvements. It is most likely that significant progress will be promoted by longer sequencing reads capabilities currently emerging in the field.

## Supporting Information

S1 Fig Relationship between ERCC rld normalized counts and their nominal concentration. (PDF)

S2 Fig Scatter plot of DESeq2 normalized ratio between ERCC mix2 and ERCC mix 1 versus the expected ratio for day 4. (PDF)

S3 Fig Scatter plots of mouse single locus spikes: expected vs observed concentrations. (PDF)

S4 Fig Abundance estimation of spikes with multiple isoforms per locus, using various bioinformatics tools. (PDF)

S5 Fig ROC analysis using p values and IVT spike-ins. (PDF)

S6 Fig Final Linear Regression Model plots for single spike-ins. (PDF)

S1 File Scotty output. (PDF)

S2 File RNA-Seq power computation. (DOCX)

S1 Table Design of spike-ins of loci with multiple transcripts. (DOCX)

S2 Table Design of spike-ins with a single transcript per locus. (DOCX)

S3 Table Description of samples. (DOCX)

S4 Table Statistics of mapping results extracted from Partek. (DOCX)

S5 Table Final Linear Regression Models using 47 spike-ins. (DOCX)

S6 Table Confidence intervals for Linear Regression Models R^2^. (DOCX)

## References

[1] Hooper JE. A survey of software for genome-wide discovery of differential splicing in RNA-Seq data. Hum Genomics. 2014;8:3 10.1186/1479-7364-8-3 24447644 PMC3903050

[2] Estivill X. Genetic variation and alternative splicing. Nat Biotechnol. 2015;33(4):357–9. 10.1038/nbt.3195 . 25850059

[3] Gabut M, Samavarchi-Tehrani P, Wang X, Slobodeniuc V, O'Hanlon D, Sung HK, et al An alternative splicing switch regulates embryonic stem cell pluripotency and reprogramming. Cell. 2011;147(1):132–46. 10.1016/j.cell.2011.08.023 . 21924763

[4] Nilsen TW, Graveley BR. Expansion of the eukaryotic proteome by alternative splicing. Nature. 2010;463(7280):457–63. 10.1038/nature08909 20110989 PMC3443858

[5] Singh P, Alley TL, Wright SM, Kamdar S, Schott W, Wilpan RY, et al Global changes in processing of mRNA 3' untranslated regions characterize clinically distinct cancer subtypes. Cancer Res. 2009;69(24):9422–30. 10.1158/0008-5472.CAN-09-2236 19934316 PMC2794997

[6] Pan Q, Shai O, Lee LJ, Frey BJ, Blencowe BJ. Deep surveying of alternative splicing complexity in the human transcriptome by high-throughput sequencing. Nat Genet. 2008;40(12):1413–5. 10.1038/ng.259 . 18978789

[7] Anders S, Reyes A, Huber W. Detecting differential usage of exons from RNA-seq data. Genome Res. 2012;22(10):2008–17. 10.1101/gr.133744.111 22722343 PMC3460195

[8] Lee Y, Rio DC. Mechanisms and Regulation of Alternative Pre-mRNA Splicing. Annu Rev Biochem. 2015 10.1146/annurev-biochem-060614-034316 . 25784052 PMC4526142

[9] Wang J, Ye Z, Huang TH, Shi H, Jin V. A survey of computational methods in transcriptome-wide alternative splicing analysis. Biomol Concepts. 2015;6(1):59–66. 10.1515/bmc-2014-0040 . 25719337 PMC5203768

[10] Feng H, Qin Z, Zhang X. Opportunities and methods for studying alternative splicing in cancer with RNA-Seq. Cancer Lett. 2013;340(2):179–91. 10.1016/j.canlet.2012.11.010 . 23196057

[11] Rapaport F, Khanin R, Liang Y, Pirun M, Krek A, Zumbo P, et al Comprehensive evaluation of differential gene expression analysis methods for RNA-seq data. Genome Biol. 2013;14(9):R95 10.1186/gb-2013-14-9-r95 . 24020486 PMC4054597

[12] Chandramohan R, Wu PY, Phan JH, Wang MD. Benchmarking RNA-Seq quantification tools. Conf Proc IEEE Eng Med Biol Soc. 2013;2013:647–50. 10.1109/EMBC.2013.6609583 . 24109770 PMC5003039

[13] Love MI, Huber W, Anders S. Moderated estimation of fold change and dispersion for RNA-seq data with DESeq2. Genome Biol. 2014;15(12):550 10.1186/PREACCEPT-8897612761307401 . 25516281 PMC4302049

[14] Li S, Łabaj PP, Zumbo P, Sykacek P, Shi W, Shi L, et al Detecting and correcting systematic variation in large-scale RNA sequencing data. Nat Biotechnol. 2014;32(9):888–95. 10.1038/nbt.3000 25150837 PMC4160374

[15] Kanitz A, Gypas F, Gruber AJ, Gruber AR, Martin G, Zavolan M. Comparative assessment of methods for the computational inference of transcript isoform abundance from RNA-seq data. Genome Biol. 2015;16:150 10.1186/s13059-015-0702-5 26201343 PMC4511015

[16] Angelini C, De Canditiis D, De Feis I. Computational approaches for isoform detection and estimation: good and bad news. BMC Bioinformatics. 2014;15:135 10.1186/1471-2105-15-135 24885830 PMC4098781

[17] Steijger T, Abril JF, Engström PG, Kokocinski F, Hubbard TJ, Guigó R, et al Assessment of transcript reconstruction methods for RNA-seq. Nat Methods. 2013;10(12):1177–84. 10.1038/nmeth.2714 24185837 PMC3851240

[18] Baker SC, Bauer SR, Beyer RP, Brenton JD, Bromley B, Burrill J, et al The External RNA Controls Consortium: a progress report. Nat Methods. 2005;2(10):731–4. 10.1038/nmeth1005-731 . 16179916

[19] Risso D, Ngai J, Speed TP, Dudoit S. Normalization of RNA-seq data using factor analysis of control genes or samples. Nat Biotechnol. 2014;32(9):896–902. 10.1038/nbt.2931 . 25150836 PMC4404308

[20] Consortium SM-I. A comprehensive assessment of RNA-seq accuracy, reproducibility and information content by the Sequencing Quality Control Consortium. Nat Biotechnol. 2014;32(9):903–14. 10.1038/nbt.2957 . 25150838 PMC4321899

[21] Trapnell C, Pachter L, Salzberg SL. TopHat: discovering splice junctions with RNA-Seq. Bioinformatics. 2009;25(9):1105–11. 10.1093/bioinformatics/btp120 19289445 PMC2672628

[22] Trapnell C, Williams BA, Pertea G, Mortazavi A, Kwan G, van Baren MJ, et al Transcript assembly and quantification by RNA-Seq reveals unannotated transcripts and isoform switching during cell differentiation. Nat Biotechnol. 2010;28(5):511–5. 10.1038/nbt.1621 20436464 PMC3146043

[23] Roberts A, Pimentel H, Trapnell C, Pachter L. Identification of novel transcripts in annotated genomes using RNA-Seq. Bioinformatics. 2011;27(17):2325–9. 10.1093/bioinformatics/btr355 . 21697122

[24] Trapnell C, Roberts A, Goff L, Pertea G, Kim D, Kelley DR, et al Differential gene and transcript expression analysis of RNA-seq experiments with TopHat and Cufflinks. Nat Protoc. 2012;7(3):562–78. 10.1038/nprot.2012.016 22383036 PMC3334321

[25] Grabherr MG, Haas BJ, Yassour M, Levin JZ, Thompson DA, Amit I, et al Full-length transcriptome assembly from RNA-Seq data without a reference genome. Nat Biotechnol. 2011;29(7):644–52. 10.1038/nbt.1883 21572440 PMC3571712

[26] Kent WJ. BLAT—the BLAST-like alignment tool. Genome Res. 2002;12(4):656–64. 10.1101/gr.229202 Article published online before March 2002. 11932250 PMC187518

[27] Smith-Unna RD, Boursnell C, Patro R, Hibberd JM, Kelly S. TransRate: reference free quality assessment of de-novo transcriptome assemblies. bioRxiv doi:10.1101/0216262015. PMC4971766 27252236

[28] Quinlan AR, Hall IM. BEDTools: a flexible suite of utilities for comparing genomic features. Bioinformatics. 2010;26(6):841–2. 10.1093/bioinformatics/btq033 20110278 PMC2832824

[29] Anders S, Pyl PT, Huber W. HTSeq-a Python framework to work with high-throughput sequencing data. Bioinformatics. 2015;31(2):166–9. 10.1093/bioinformatics/btu638 . 25260700 PMC4287950

[30] Trapnell C, Hendrickson DG, Sauvageau M, Goff L, Rinn JL, Pachter L. Differential analysis of gene regulation at transcript resolution with RNA-seq. Nat Biotechnol. 2013;31(1):46–53. 10.1038/nbt.2450 . 23222703 PMC3869392

[31] Anders S, Huber W. Differential expression analysis for sequence count data. Genome Biol. 2010;11(10):R106 10.1186/gb-2010-11-10-r106 20979621 PMC3218662

[32] Anders S, McCarthy DJ, Chen Y, Okoniewski M, Smyth GK, Huber W, et al Count-based differential expression analysis of RNA sequencing data using R and Bioconductor. Nat Protoc. 2013;8(9):1765–86. 10.1038/nprot.2013.099 . 23975260

[33] Roberts A, Trapnell C, Donaghey J, Rinn JL, Pachter L. Improving RNA-Seq expression estimates by correcting for fragment bias. Genome Biol. 2011;12(3):R22 10.1186/gb-2011-12-3-r22 21410973 PMC3129672

[34] Li B, Dewey CN. RSEM: accurate transcript quantification from RNA-Seq data with or without a reference genome. BMC Bioinformatics. 2011;12:323 10.1186/1471-2105-12-323 21816040 PMC3163565

[35] Langmead B, Trapnell C, Pop M, Salzberg SL. Ultrafast and memory-efficient alignment of short DNA sequences to the human genome. Genome Biol. 2009;10(3):R25 10.1186/gb-2009-10-3-r25 19261174 PMC2690996

[36] Hansen KD, Irizarry RA, Wu Z. Removing technical variability in RNA-seq data using conditional quantile normalization. Biostatistics. 2012;13(2):204–16. 10.1093/biostatistics/kxr054 22285995 PMC3297825

[37] Robin X, Turck N, Hainard A, Tiberti N, Lisacek F, Sanchez JC, et al pROC: an open-source package for R and S+ to analyze and compare ROC curves. BMC Bioinformatics. 2011;12:77 10.1186/1471-2105-12-77 21414208 PMC3068975

[38] Busby MA, Stewart C, Miller CA, Grzeda KR, Marth GT. Scotty: a web tool for designing RNA-Seq experiments to measure differential gene expression. Bioinformatics. 2013;29(5):656–7. 10.1093/bioinformatics/btt015 23314327 PMC3582267

[39] Hart SN, Therneau TM, Zhang Y, Poland GA, Kocher JP. Calculating sample size estimates for RNA sequencing data. J Comput Biol. 2013;20(12):970–8. 10.1089/cmb.2012.0283 23961961 PMC3842884

[40] Leng N, Dawson JA, Thomson JA, Ruotti V, Rissman AI, Smits BM, et al EBSeq: an empirical Bayes hierarchical model for inference in RNA-seq experiments. Bioinformatics. 2013;29(8):1035–43. 10.1093/bioinformatics/btt087 23428641 PMC3624807

[41] Ken K, Keke L. Package ‘MBESS’. 2012.

[42] Xu J, Wang H, Liang T, Cai X, Rao X, Huang Z, et al Retinoic acid promotes neural conversion of mouse embryonic stem cells in adherent monoculture. Mol Biol Rep. 2012;39(2):789–95. 10.1007/s11033-011-0800-8 . 21611753

[43] Roberts A, Pachter L. Streaming fragment assignment for real-time analysis of sequencing experiments. Nat Methods. 2013;10(1):71–3. 10.1038/nmeth.2251 . 23160280 PMC3880119

