# Supplementary figures and images for: Using Synthetic Mouse Spike-In Transcripts to Evaluate RNA-Seq Analysis Tools
Source: PLoS One. 2016 Apr 21;11(4):e0153782. doi: 10.1371/journal.pone.0153782 (PMC4839710; doi:10.1371/journal.pone.0153782)

Fig. S1 Relationship between ERCC rld normalized counts and their nominal concentration

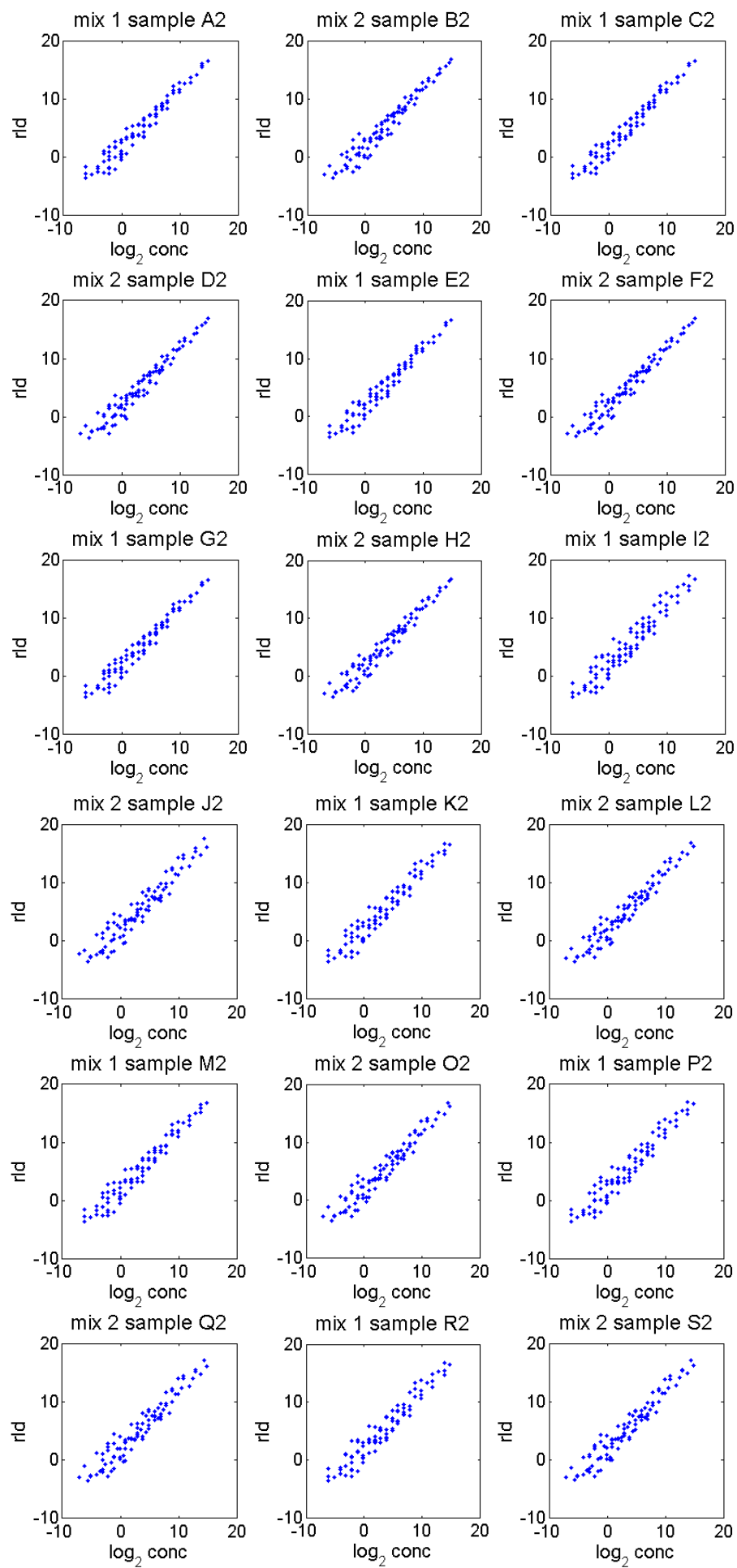

Supplement: S1 Fig — (PDF) [file pone.0153782.s001.pdf]

Fig. 5. ROC analysis using p values and IVT spike-ins (RefSeq+ annotation)

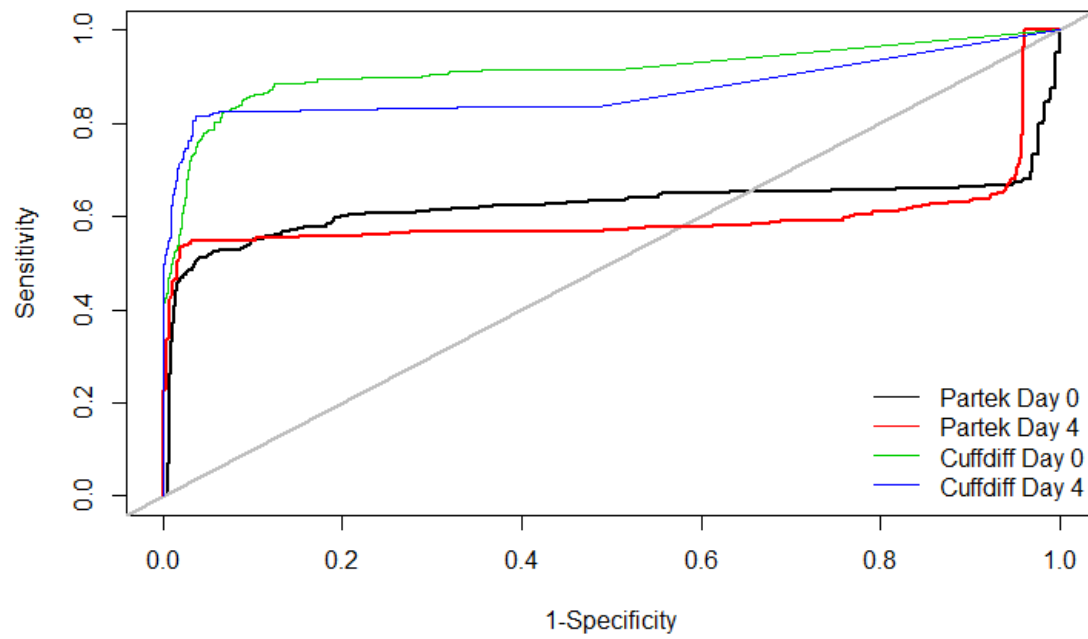

Supplement: S5 Fig — (PDF) [file pone.0153782.s005.pdf]
