# Supplementary material for: Using Synthetic Mouse Spike-In Transcripts to Evaluate RNA-Seq Analysis Tools
Source: PLoS One. 2016 Apr 21;11(4):e0153782. doi: 10.1371/journal.pone.0153782 (PMC4839710; doi:10.1371/journal.pone.0153782)
Supplement: S3 Fig — (PDF) [file pone.0153782.s003.pdf]

Fig. S3 Scatter plots of mouse single locus spikes: expected vs. observed concentrations

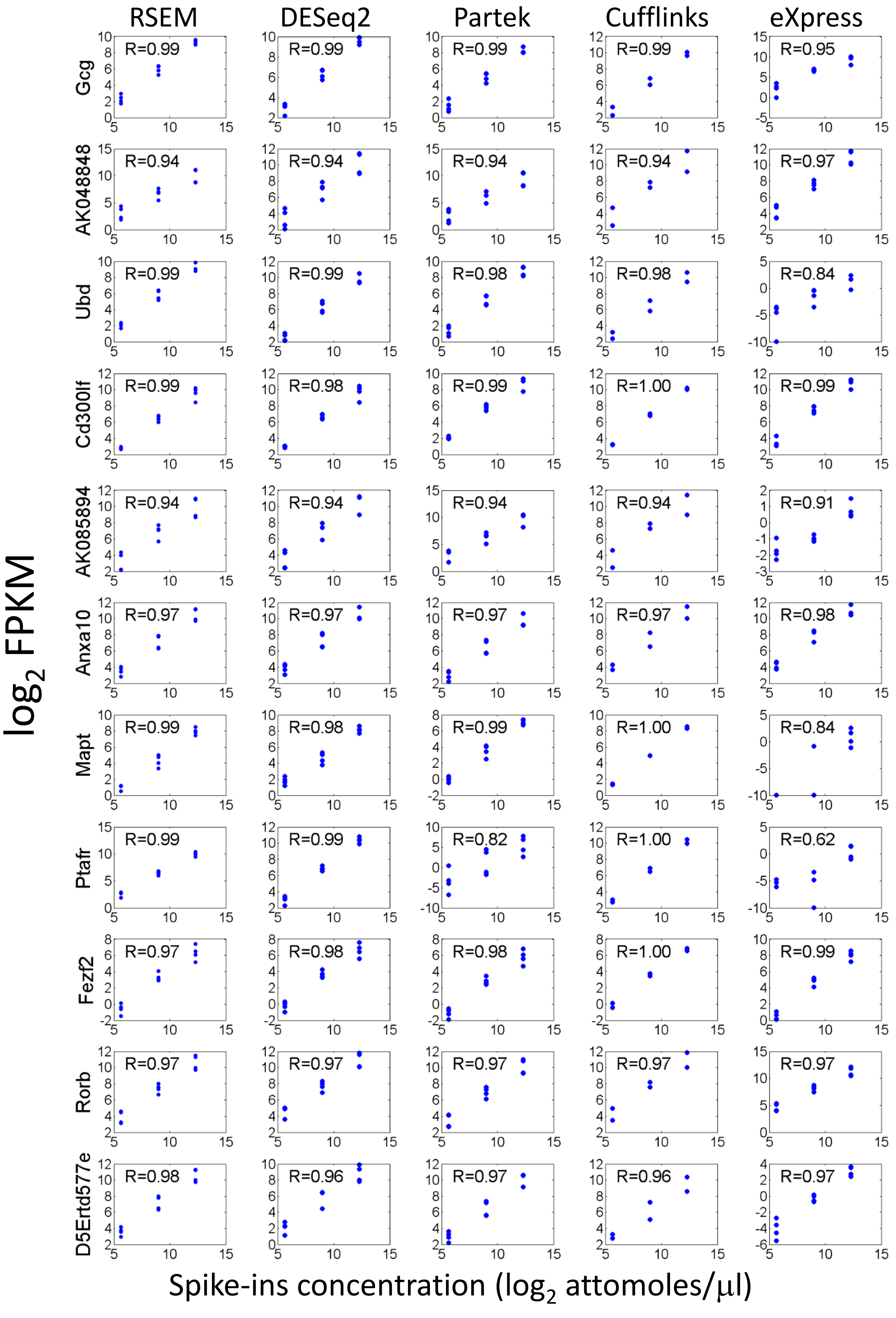

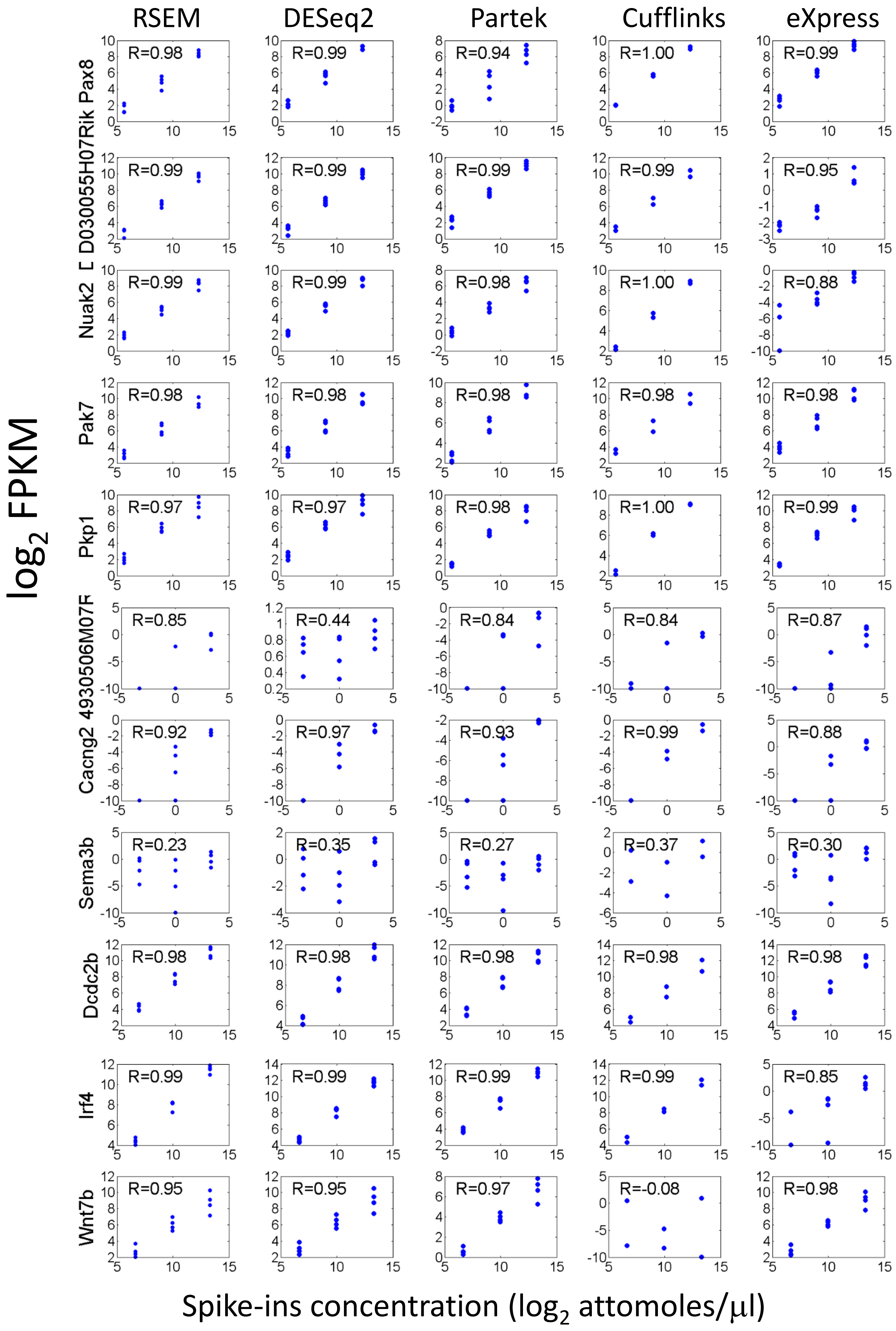

RSEM
DESeq2
Partek
Cufflinks
eXpress
Pax8
D030055H07Rik
Nuak2
Pak7
Pkp1
4930506M07F
Cacng2
Sema3b
Dcdc2b
Irf4
Wnt7b
R=0.98
R=0.99
R=0.94
R=1.00
R=0.99
R=0.99
R=0.99
R=0.99
R=0.99
R=0.95
R=0.99
R=0.99
R=0.98
R=1.00
R=0.88
R=0.98
R=0.98
R=0.98
R=0.98
R=0.98
R=0.97
R=0.97
R=0.98
R=1.00
R=0.99
R=0.85
R=0.44
R=0.84
R=0.84
R=0.87
R=0.92
R=0.97
R=0.93
R=0.99
R=0.88
R=0.23
R=0.35
R=0.27
R=0.37
R=0.30
R=0.98
R=0.98
R=0.98
R=0.98
R=0.98
R=0.99
R=0.99
R=0.99
R=0.99
R=0.85
R=0.95
R=0.95
R=0.97
R=-0.08
R=0.98
log2 FPKM
Spike-ins concentration (log2 attomoles/μl)
