# Supplementary material for: Using Synthetic Mouse Spike-In Transcripts to Evaluate RNA-Seq Analysis Tools
Source: PLoS One. 2016 Apr 21;11(4):e0153782. doi: 10.1371/journal.pone.0153782 (PMC4839710; doi:10.1371/journal.pone.0153782)

Fig S4. Abundance estimation of spikes with multiple isoforms per locus, using various bioinformatics tools

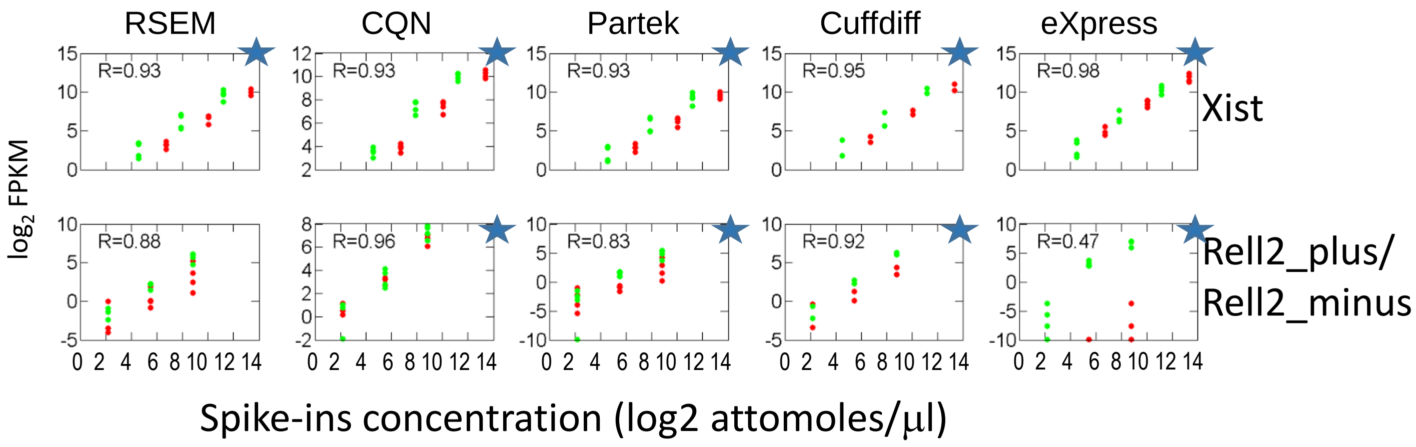

Supplement: S4 Fig — (PDF) [file pone.0153782.s004.pdf]
