# Supplementary material for: Using Synthetic Mouse Spike-In Transcripts to Evaluate RNA-Seq Analysis Tools
Source: PLoS One. 2016 Apr 21;11(4):e0153782. doi: 10.1371/journal.pone.0153782 (PMC4839710; doi:10.1371/journal.pone.0153782)

Fig. S6. Final Linear Regression Model plots with single spike-ins

Plot A shows expected spike-in values versus DESeq2-HTSeq model observed values.

Plot B shows expected spike-in values versus CQN-HTSeq model observed values.

A.

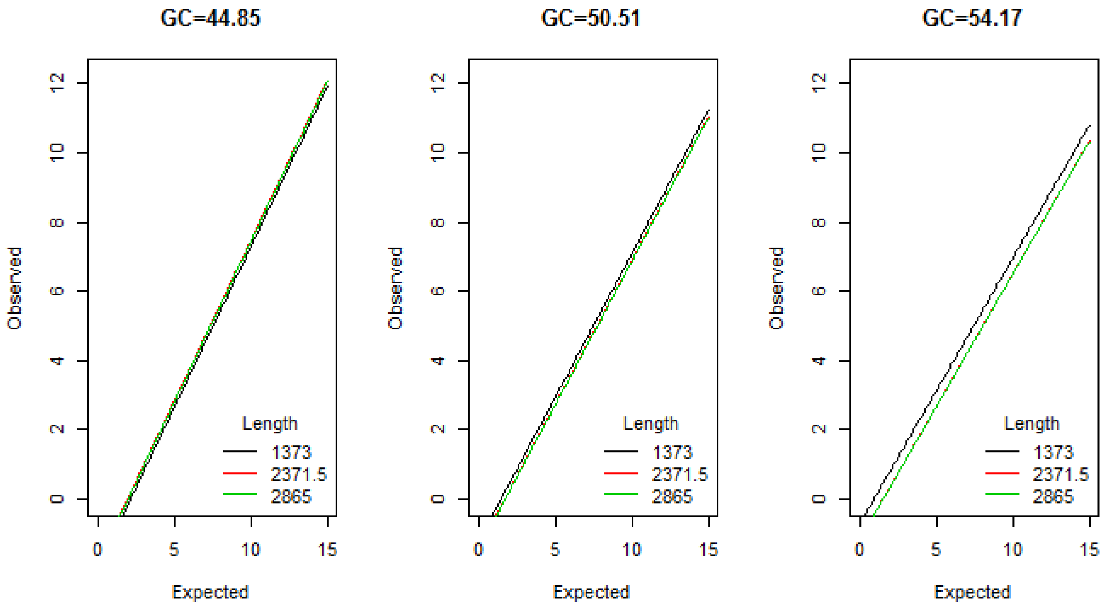

B.

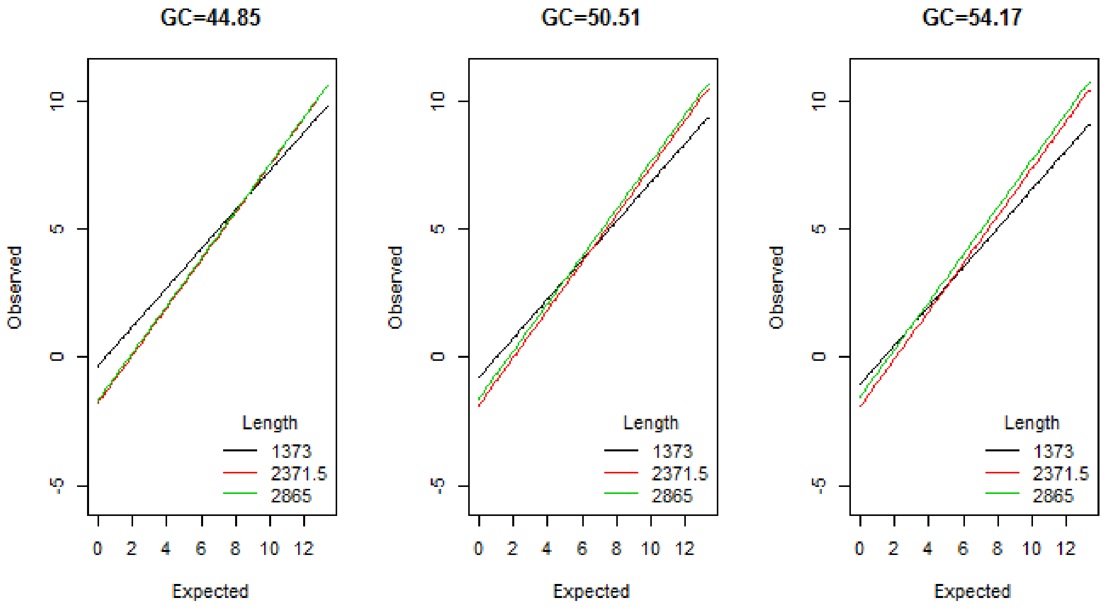

Supplement: S6 Fig — (PDF) [file pone.0153782.s006.pdf]
