# Supplementary material for: Using Synthetic Mouse Spike-In Transcripts to Evaluate RNA-Seq Analysis Tools
Source: PLoS One. 2016 Apr 21;11(4):e0153782. doi: 10.1371/journal.pone.0153782 (PMC4839710; doi:10.1371/journal.pone.0153782)
Supplement: S2 File — (DOCX) [file pone.0153782.s008.docx]

S2 File. RNA-Seq power computation

|  |  | Depth | | | | | | | | | | | | | | | | | | |
| --- | --- | --- | --- | --- | --- | --- | --- | --- | --- | --- | --- | --- | --- | --- | --- | --- | --- | --- | --- | --- |
| CV | Effect | 5 | 6 | 7 | 8 | 9 | 10 | 11 | 12 | 13 | 14 | 15 | 16 | 17 | 18 | 19 | 20 | 100 | 1000 | 5000 |
| 0.5 | 10 | 2 | 2 | 2 | 2 | 2 | 2 | 2 | 1 | 1 | 1 | 1 | 1 | 1 | 1 | 1 | 1 | 1 | 1 | 1 |
| 0.5 | 100 | 1 | 1 | 1 | 1 | 1 | 1 | 1 | 1 | 1 | 1 | 1 | 1 | 1 | 1 | 1 | 1 | 1 | 1 | 1 |
| 0.75 | 10 | 3 | 3 | 3 | 3 | 2 | 2 | 2 | 2 | 2 | 2 | 2 | 2 | 2 | 2 | 2 | 2 | 2 | 2 | 2 |
| 0.75 | 100 | 1 | 1 | 1 | 1 | 1 | 1 | 1 | 1 | 1 | 1 | 1 | 1 | 1 | 1 | 1 | 1 | 1 | 1 | 1 |
| 1 | 10 | 4 | 4 | 4 | 4 | 4 | 4 | 4 | 4 | 4 | 4 | 4 | 4 | 4 | 4 | 4 | 4 | 3 | 3 | 3 |
| 1 | 100 | 1 | 1 | 1 | 1 | 1 | 1 | 1 | 1 | 1 | 1 | 1 | 1 | 1 | 1 | 1 | 1 | 1 | 1 | 1 |

RNASeqPower was run with alpha=0.05, power=0.8, the effect size selected was the incremental used in the experiment (10 and 100), CV values and depth selected are depicted in the table above. Using our replicate spike-in expression values (Partek rpkm), CV of 0.75 or lower contains 85% of our spike-ins within each sample type (which is a combination of day and mix type). From this table we can conclude that 2 replicates are sufficient in most cases.

Commands used:

n1<-ceiling(rnapower(depth=c(5:20,100,1000,5000),cv=.5,cv2=.5,effect=10,alpha=0.05,power=0.8))

n2<-ceiling(rnapower(depth=c(5:20,100,1000,5000),cv=.75,cv2=.75,effect=10,alpha=0.05,power=0.8))

n3<-ceiling(rnapower(depth=c(5:20,100,1000,5000),cv=1,cv2=1,effect=10,alpha=0.05,power=0.8))

nm<-rbind(n1,n2,n3)

dimnames(nm)<-list(as.character(c(0.5,0.75,1)),c(5:20,100,1000,5000))
