# Supplementary material for: Using Synthetic Mouse Spike-In Transcripts to Evaluate RNA-Seq Analysis Tools
Source: PLoS One. 2016 Apr 21;11(4):e0153782. doi: 10.1371/journal.pone.0153782 (PMC4839710; doi:10.1371/journal.pone.0153782)
Supplement: S1 Table — (DOCX) [file pone.0153782.s009.docx]

Table S1. Design of spike-ins of loci with multiple transcripts (mix concentrations are attomoles/μl)

| **spiked-in category** | **locus number** | **transcript accession** | **Forchheimer serial #** | **gene id** | **gene_id in our refseq gtf** | **gene_id in our gencode gtf** | **Vector type** | **Restriction** | **strand** | **Length** | **%GC** | **Mix1** | **Mix2** | **Mix3** |  |
| --- | --- | --- | --- | --- | --- | --- | --- | --- | --- | --- | --- | --- | --- | --- | --- |
| **locus analysis** | 1 | AK006256 | 1082 | Dnali1 | Dnali1_minus | Dnali1 | Bluescript | SacI | - | 973 | 57 | 10.0 | 100.0 | 1000.0 | attomoles/ul |
|  | 1 | AK077109 | 1083 | Dnali1 | Dnali1_minus | Dnali1 | l-FLC1 | BamHI | - | 2239 | 52 | 1.5 | 15.0 | 150.0 | 15% -second transcript |
|  | 2 | AK030714 | 1084 | Pomc | Pomc_plus | Pomc | l-FLC1 | BamHI | + | 1195 | 57 | 1000.0 | 10.0 | 100.0 |  |
|  | 2 | AK017581 | 1085 | Pomc | Pomc_plus | Pomc | l-FLC1 | BamHI | + | 1130 | 58 | 150.0 | 1.5 | 15.0 |  |
|  | 2 | AK017492 | 1086 | Pomc | Pomc_plus | Pomc | l-FLC1 | BamHI | + | 1160 | 58 | 150.0 | 1.5 | 15.0 |  |
|  | 3 | AK015340 | 1178 | C8b | C8b_plus | C8b | l-FLC1 | BamHI | + | 1214 | 49 | 100.0 | 1000.0 | 10.0 |  |
|  | 3 | AK050313 | 1177 | C8b | C8b_plus | C8b | l-FLC1 | BamHI | + | 2392 | 48 | 15.0 | 150.0 | 1.5 |  |
|  | 4 | AK044762 | 1168 | Rasa4 | Rasa4_plus | Rasa4 | l-FLC1 | BamHI | + | 2836 | 57 | 10.0 | 100.0 | 1000.0 | attomoles/ul |
|  | 4 | AK029206 | 1169 | Rasa4 | Rasa4_plus | Rasa4 | l-FLC1 | BamHI | + | 1623 | 54 | 3.0 | 30.0 | 300.0 | 30% -second transcript |
|  | 5 | AK008207 | 1170 | Acbd4 | Acbd4_plus | Acbd4 | Bluescript | SacI | + | 1233 | 58 | 1000.0 | 10.0 | 100.0 |  |
|  | 5 | AK079604 | 1172 | Acbd4 | Acbd4_plus | Acbd4 | l-FLC1 | BamHI | + | 2099 | 58 | 300.0 | 3.0 | 30.0 |  |
|  | 6 | AK079344 | 1181 | Fam83f | Fam83f_plus | Fam83f | l-FLC1 | BamHI | + | 2477 | 57 | 100.0 | 1000.0 | 10.0 |  |
|  | 6 | AK030342 | 1179 | Fam83f | Fam83f_plus | Fam83f | l-FLC1 | BamHI | + | 3125 | 57 | 30.0 | 300.0 | 3.0 |  |
|  | 6 | AK038037 | 1182 | A430088P11Rik | A430088P11Rik_minus | A430088P11Rik | l-FLC1 | ApaI | - | 3096 | 49 | 10.0 | 100.0 | 1000.0 |  |
|  | 7 | AK045632 | 1188 | Slc1a1 | Slc1a1_plus | Slc1a1 | l-FLC1 | BamHI | + | 1915 | 51 | 1000.0 | 100.0 | 10.0 |  |
|  | 7 | AK016454 | 1209 | 4430402I18Rik | AK016454_minus | 4430402I18Rik | l-FLC1 | BamHI | - | 1965 | 48 | 300.0 | 30.0 | 3.0 |  |
|  | 8 | AK044097 | 1183 | Rell2 | Rell2_plus | Rell2 | l-FLC1 | BamHI | + | 1867 | 60 | 4.5 | 45.0 | 450.0 | atomoles/ul |
|  | 8 | AK014510 | 1180 | Rell2 | Rell2_minus | Rell2 | l-FLC1 | BamHI | - | 1475 | 54 | 4.5 | 45.0 | 450.0 | 45% -second transcript |
|  | 9 | AK045706 | 1201 | Jph3 | Jph3_plus | Jph3 | l-FLC1 | BamHI | + | 3776 | 60 | 1000.0 | 10.0 | 100.0 |  |
|  | 9 | AK046136 | 1212 | Jph3 | Jph3_plus | Jph3 | i | BamHI | + | 3005 | 60 | 450.0 | 4.5 | 45.0 |  |
|  | 10 | AK035433 | 1213 | A430018G15Rik | A430018G15Rik_plus | A430018G15Rik | l-FLC1 | BamHI | + | 1467 | 39 | 100.0 | 1000.0 | 10.0 |  |
|  | 10 | AK079699 | 1223 | A430018G15Rik | A430018G15Rik_plus | A430018G15Rik | l-FLC1 | BamHI | + | 919 | 49 | 45.0 | 450.0 | 4.5 |  |
|  | 10 | AK038633 | 1222 | Cacnb4 | Cacnb4_minus | Cacnb4 | l-FLC1 | BamHI | - | 2477 | 46 | 45.0 | 450.0 | 4.5 |  |
