# Supplementary material for: Using Synthetic Mouse Spike-In Transcripts to Evaluate RNA-Seq Analysis Tools
Source: PLoS One. 2016 Apr 21;11(4):e0153782. doi: 10.1371/journal.pone.0153782 (PMC4839710; doi:10.1371/journal.pone.0153782)
Supplement: S2 Table — (DOCX) [file pone.0153782.s010.docx]

Table S2. Design of spike-ins with a single transcript per locus (mix concentrations are attomoles/μl)

| **spiked-in category** | locus number | transcript accession | Forchheimer serial # | gene id | gene_id in our gtf | gene_id in our gencode refseq | Vector type | Restriction | strand | Length | %GC | Mix1 | Mix2 | Mix3 |
| --- | --- | --- | --- | --- | --- | --- | --- | --- | --- | --- | --- | --- | --- | --- |
| **GC length analysis** | 12 | AK007911 | 756 | Gcg | Gcg_minus | Gcg | Bluescript | SacI | - | 923 | 48 | 50.0 | 500.0 | 5000.0 |
|  | 13 | AK048848 | 191 |  | AK048848_minus | AK048848 | l-FLC1 | BamHI | - | 975 | 33 | 5000.0 | 50.0 | 500.0 |
|  | 14 | AK008552 | 980 | Ubd | Ubd_plus | Ubd | Bluescript | SacI | + | 999 | 44 | 500.0 | 5000.0 | 50.0 |
|  | 15 | AK089313 | 1187 | Cd300lf | Cd300lf_minus | Cd300lf | l-FLC1 | DraII | - | 1212 | 53 | 50.0 | 500.0 | 5000.0 |
|  | 16 | AK085894 | 437 |  | AK085894_plus | AK085894 | l-FLC1 | BamHI | + | 1345 | 35 | 5000.0 | 50.0 | 500.0 |
|  | 17 | AK020288 | 1199 | Anxa10 | Anxa10_minus | Anxa10 | l-FLC1 | BamHI | - | 1373 | 43 | 500.0 | 5000.0 | 50.0 |
|  | 11 | AK039861 | 1166 | Xist | Xist_minus | Xist | l-FLC1 | ApaI | - | 1686 | 50 | 10682.4 | 106.8 | 1068.2 |
|  | 18 | AK078167 | 485 | Mapt | Mapt_plus | Mapt | l-FLC1 | DraII | + | 1715 | 54 | 5000.0 | 50.0 | 500.0 |
|  | 19 | AK032547 | 132 | Ptafr | Ptafr_plus | Ptafr | l-FLC1 | BamHI | + | 1810 | 51 | 500.0 | 5000.0 | 50.0 |
|  | 20 | AK019996 | 958 | Fezf2 | Fezf2_minus | Fezf2 | l-FLC1 | BamHI | - | 2210 | 56 | 50.0 | 500.0 | 5000.0 |
|  | 21 | AK044421 | 129 | Rorb | Rorb_minus | Rorb | l-FLC1 | KpnI | - | 2533 | 45 | 5000.0 | 50.0 | 500.0 |
|  | 22 | AK054417 | 1014 | D5Ertd577e | D5Ertd577e_plus | D5Ertd577e | l-FLC1 | BamHI | + | 2629 | 44 | 500.0 | 5000.0 | 50.0 |
|  | 24 | AK050717 | 480 | Pax8 | Pax8_minus | Pax8 | l-FLC1 | BamHI | - | 2685 | 54 | 5000.0 | 50.0 | 500.0 |
|  | 25 | AK051019 | 1225 | D030055H07Rik | D030055H07Rik_plus | D030055H07Rik | l-FLC1 | BamHI | + | 2865 | 50 | 500.0 | 5000.0 | 50.0 |
|  | 26 | AK033672 | 410 | Nuak2 | Nuak2_plus | Nuak2 | l-FLC1 | BamHI | + | 3246 | 54 | 50.0 | 500.0 | 5000.0 |
|  | 11 | AK051106 | 1167 | Xist | Xist_minus | Xist | l-FLC1 | ApaI | - | 3611 | 40 | 23.4 | 234.0 | 2340.3 |
|  | 27 | AK032593 | 169 | Pak7 | Pak7_minus | Pak7 | l-FLC1 | ApaI | - | 4126 | 47 | 500.0 | 5000.0 | 50.0 |
|  | 28 | AK036856 | 399 | Pkp1 | Pkp1_minus | Pkp1 | l-FLC1 | KpnI | - | 4178 | 55 | 50.0 | 500.0 | 5000.0 |
| **range** | 29 | AK015721 | 195 | 4930506M07Rik | 4930506M07Rik_minus | 4930506M07Rik | l-FLC1 | ApaI | - | 1425 | 50 | 0.1 | 1.0 | 10.0 |
|  | 30 | AK045362 | 1216 | Cacng2 | Cacng2_minus | Cacng2 | l-FLC1 | BamHI | - | 2579 | 55 | 1.0 | 10.0 | 0.1 |
|  | 31 | AK037034 | 246 | Sema3b | Sema3b_minus | Sema3b | l-FLC1 | ApaI | - | 3482 | 57 | 10.0 | 0.1 | 1.0 |
|  | 32 | AK086820 | 127 | Dcdc2b | Dcdc2b_minus | Dcdc2b | l-FLC1 | BamHI | - | 1480 | 48 | 10000.0 | 1000.0 | 100.0 |
|  | 33 | AK089819 | 981 | Irf4 | Irf4_plus | Irf4 | l-FLC1 | BamHI | + | 2626 | 51 | 100.0 | 10000.0 | 1000.0 |
|  | 34 | AK087499 | 641 | Wnt7b | Wnt7b_minus | Wnt7b | l-FLC1 | BamHI | - | 3551 | 59 | 1000.0 | 100.0 | 10000.0 |
