# Supplementary material for: Using Synthetic Mouse Spike-In Transcripts to Evaluate RNA-Seq Analysis Tools
Source: PLoS One. 2016 Apr 21;11(4):e0153782. doi: 10.1371/journal.pone.0153782 (PMC4839710; doi:10.1371/journal.pone.0153782)
Supplement: S3 Table — (DOCX) [file pone.0153782.s011.docx]

Table S3. Description of samples

| **RNA type** | **Replicate** | **ERCC mix** | **IVT Spike-ins mix** | **Supplied samples** |
| --- | --- | --- | --- | --- |
| Day 0 | 1 | 1 | mix1 | C |
| Day 0 | 1 | 2 | mix2 | D |
| Day 0 | 1 | 1 | mix3 | E |
| Day 0 | 1 | 2 | mix4 | F |
| Day 0 | 2 | 1 | mix1 | G |
| Day 0 | 2 | 2 | mix2 | H |
| Day 0 | 2 | 1 | mix3 | I |
| Day 0 | 2 | 2 | mix4 | J |
| Day 4 | 1 | 1 | mix1 | K |
| Day 4 | 1 | 2 | mix2 | L |
| Day 4 | 1 | 1 | mix3 | M |
| Day 4 | 1 | 2 | mix4 | O |
| Day 4 | 2 | 1 | mix1 | P |
| Day 4 | 2 | 2 | mix2 | Q |
| Day 4 | 2 | 1 | mix3 | R |
| Day 4 | 2 | 2 | mix4 | S |
