# Supplementary material for: Using Synthetic Mouse Spike-In Transcripts to Evaluate RNA-Seq Analysis Tools
Source: PLoS One. 2016 Apr 21;11(4):e0153782. doi: 10.1371/journal.pone.0153782 (PMC4839710; doi:10.1371/journal.pone.0153782)
Supplement: S4 Table — (DOCX) [file pone.0153782.s012.docx]

Table S4. Statistics of mapping results extracted from Partek

| Sample ID | Number of Alignments | RN type | Spikes mix | Total Number of Reads | Percentage of reads which fully overlap exon | Percentage of paired-end reads where one alignment fully overlaps an exonic region but not compatible with any transcript | Percentage of reads which partially overlap exon | Percentage of reads within an intron | Percentage of reads between genes | Total number of transcripts in the model | Percentage of transcripts with reads | Total reads with junctions | Reads with junctions that are compatible with a transcript |
| --- | --- | --- | --- | --- | --- | --- | --- | --- | --- | --- | --- | --- | --- |
| C2 | 100,247,863.0 | RAday0 | mix1 | 38,119,032.0 | 61.3 | 9.1 | 1.4 | 11.0 | 17.3 | 33,753.0 | 72.9 | 12,054,016.0 | 10,465,939.0 |
| D2 | 109,359,303.0 | RAday0 | mix2 | 41,606,362.0 | 61.8 | 8.7 | 1.4 | 11.0 | 17.1 | 33,753.0 | 73.3 | 13,310,082.0 | 11,639,026.0 |
| E2 | 108,181,972.0 | RAday0 | mix3 | 41,425,742.0 | 61.4 | 9.2 | 1.4 | 10.9 | 17.1 | 33,753.0 | 73.1 | 13,381,650.0 | 11,648,665.0 |
| F2 | 94,854,385.0 | RAday0 | mix4 | 36,198,461.0 | 61.4 | 8.4 | 1.4 | 11.1 | 17.6 | 33,753.0 | 72.7 | 11,505,273.0 | 10,125,031.0 |
| G2 | 102,445,511.0 | RAday0 | mix1 | 39,680,681.0 | 61.9 | 9.0 | 1.4 | 11.8 | 15.8 | 33,753.0 | 73.1 | 12,611,213.0 | 11,000,119.0 |
| H2 | 132,649,775.0 | RAday0 | mix2 | 51,640,047.0 | 62.5 | 8.0 | 1.4 | 12.8 | 15.4 | 33,753.0 | 73.9 | 15,975,767.0 | 14,083,577.0 |
| I2 | 129,273,982.0 | RAday0 | mix3 | 51,574,715.0 | 60.4 | 6.5 | 1.5 | 16.4 | 15.2 | 33,753.0 | 71.7 | 12,200,199.0 | 10,778,482.0 |
| J2 | 118,942,433.0 | RAday0 | mix4 | 47,239,502.0 | 60.4 | 6.3 | 1.5 | 16.5 | 15.4 | 33,753.0 | 71.7 | 11,738,017.0 | 10,437,020.0 |
| K2 | 105,374,769.0 | RAday4 | mix1 | 42,296,469.0 | 63.4 | 7.1 | 1.0 | 10.5 | 18.1 | 33,753.0 | 73.7 | 9,921,688.0 | 8,741,488.0 |
| L2 | 111,200,723.0 | RAday4 | mix2 | 43,816,118.0 | 64.3 | 7.2 | 1.1 | 10.0 | 17.5 | 33,753.0 | 74.7 | 11,893,670.0 | 10,467,322.0 |
| M2 | 121,390,508.0 | RAday4 | mix3 | 47,961,588.0 | 64.1 | 7.4 | 1.1 | 10.1 | 17.3 | 33,753.0 | 75.4 | 12,969,454.0 | 11,395,295.0 |
| O2 | 110,338,894.0 | RAday4 | mix4 | 43,722,618.0 | 63.8 | 6.9 | 1.0 | 10.3 | 17.9 | 33,753.0 | 74.4 | 11,621,609.0 | 10,295,848.0 |
| P2 | 97,249,350.0 | RAday4 | mix1 | 38,583,214.0 | 63.0 | 7.2 | 1.0 | 11.1 | 17.7 | 33,753.0 | 73.6 | 9,384,150.0 | 8,279,469.0 |
| Q2 | 90,174,358.0 | RAday4 | mix2 | 35,727,480.0 | 63.5 | 6.7 | 1.0 | 11.0 | 17.8 | 33,753.0 | 73.2 | 8,715,919.0 | 7,739,651.0 |
| R2 | 98,585,080.0 | RAday4 | mix3 | 39,025,046.0 | 63.1 | 6.7 | 1.0 | 11.3 | 17.9 | 33,753.0 | 73.2 | 9,196,333.0 | 8,121,305.0 |
| S2 | 101,755,854.0 | RAday4 | mix4 | 40,075,882.0 | 63.0 | 6.4 | 1.0 | 11.1 | 18.5 | 33,753.0 | 74.2 | 10,064,841.0 | 8,945,939.0 |
