# Supplementary material for: Using Synthetic Mouse Spike-In Transcripts to Evaluate RNA-Seq Analysis Tools
Source: PLoS One. 2016 Apr 21;11(4):e0153782. doi: 10.1371/journal.pone.0153782 (PMC4839710; doi:10.1371/journal.pone.0153782)
Supplement: S5 Table — (DOCX) [file pone.0153782.s013.docx]

Table 5. Final Additional Linear Regression Models

| 1. **Model using normalized CQN-HTSeq FPKM values for single spikes** | | | |  |
| --- | --- | --- | --- | --- |
|  |  |  |  |  |
|  | Estimate | Std. Error | t value | Pr(>\|t\|) |
| (Intercept) | 6.89 | 1.45 | 4.76 | 0 |
| expected | 0.76 | 0.06 | 13.87 | 0 |
| day4 | -0.40 | 0.34 | -1.21 | 0.22817 |
| %GC | -0.16 | 0.03 | -5.96 | 0 |
| length/1000 | -3.38 | 0.69 | -4.89 | 0 |
| length_sq/1e+06 | 1.10 | 0.23 | 4.88 | 0 |
| expected:day4 | 0.09 | 0.04 | 2.50 | 0.01303 |
| expected:(length/1000) | 0.07 | 0.02 | 3.30 | 0.00109 |
| %GC:(length/1000) | 0.06 | 0.01 | 4.93 | 0 |
| expected:(length_sq/1e+06) | -0.12 | 0.02 | -4.95 | 0 |
|  |  |  |  |  |
| RMSE 1.12 |  |  |  |  |
| Adjusted R-squared: 0.9 | | | | |

1. **Model using normalized CQN-RSEM FPKM values for all spikes**

|  | | Estimate | Std. Error | t value | Pr(>\|t\|) |  |
| --- | --- | --- | --- | --- | --- | --- |
| (Intercept) | | 3.00 | 1.11 | 2.69 | 0.73190 |  |
| expected | | 1.05 | 0.02 | 49.89 | 0 |  |
| day4 | | 0.03 | 0.18 | 0.16 | 0.87092 |  |
| %GC | | -0.17 | 0.02 | -7.83 | 0.00000 |  |
| length/1000 | | 0.93 | 0.06 | 15.99 | 0 |  |
| length_sq/1e+06 | | -0.95 | 0.48 | -1.97 | 0.04899 |  |
| spiked_loci | | -2.67 | 0.52 | -5.18 | 0.00000 |  |
| isoforms_loci | | -29.08 | 4.75 | -6.12 | 0.00000 |  |
| expected:day4 | | 0.07 | 0.02 | 3.06 | 0.00236 |  |
| expected:(length_sq/1e+06) | | -0.04 | 0.02 | -2.79 | 0.00545 |  |
| expected:isoforms_loci | | -0.14 | 0.05 | -2.94 | 0.00341 |  |
| %GC:(length_sq/1e+06) | | 0.03 | 0.01 | 3.25 | 0.00123 |  |
| %GC:spiked_loci | | 0.07 | 0.01 | 6.51 | 0.00000 |  |
| %GC:isoforms_loci | | 0.47 | 0.09 | 5.36 | 0.00000 |  |
| (length/1000):isoforms_loci | | 3.30 | 0.33 | 10.03 | 0 |  |
| (length_sq/1e+06):spiked_loci | | -0.40 | 0.11 | -3.78 | 0.00017 |  |
| (length_sq/1e+06):isoforms_loci | | -4.00 | 0.68 | -5.91 | 0.00000 |  |
| RMSE 0.95 |  |  |  |  |  |  |
| Adjusted R-squared: 0.94 | | | | | | |
