# Supplementary material for: Using Synthetic Mouse Spike-In Transcripts to Evaluate RNA-Seq Analysis Tools
Source: PLoS One. 2016 Apr 21;11(4):e0153782. doi: 10.1371/journal.pone.0153782 (PMC4839710; doi:10.1371/journal.pone.0153782)
Supplement: S6 Table — (DOCX) [file pone.0153782.s014.docx]

Table S6. Confidence intervals for Linear Regression Models R^2^

|  | Model | 95% Lower Conf. Limit for R2 adj | 95% Upper Conf. Limit for R2 adj | R^2^ adj. |
| --- | --- | --- | --- | --- |
| 1 | Table 3A HTSeq-DESeq single spikes full | 0.89 | 0.92 | 0.91 |
| 2 | HTSeq-DESeq single spikes basic | 0.85 | 0.89 | 0.87 |
| 3 | Table 3B HTSeq-CQN single spikes full | 0.87 | 0.91 | 0.90 |
| 4 | HTSeq-CQN single spikes basic | 0.83 | 0.88 | 0.86 |
| 5 | Table S5A RSEM all spikes full | 0.93 | 0.94 | 0.94 |
| 6 | Table S5A RSEM all spikes basic | 0.83 | 0.86 | 0.85 |
| 7 | Table S5B RSEM all spikes basic and spike-ins attributes | 0.91 | 0.93 | 0.92 |
| 8 | Table S5B RSEM-CQN all spikes full | 0.93 | 0.95 | 0.94 |
| 9 | Table S5B RSEM-CQN all spikes basic | 0.83 | 0.87 | 0.85 |
| 10 | Table S5B RSEM-CQN all spikes basic and spike-ins attributes | 0.91 | 0.93 | 0.92 |
